# A yeast-based genomic strategy highlights the cell protein networks altered by FTase inhibitor peptidomimetics

**DOI:** 10.1186/1476-4598-9-197

**Published:** 2010-07-23

**Authors:** Giampiero Porcu, Cathal Wilson, Daniele Di Giandomenico, Antonella Ragnini-Wilson

**Affiliations:** 1 Department of Biology, University of Rome "Tor Vergata", Italy; 2 Translational and Cellular Pharmacology, Consorzio Mario negri Sud, S.M Imbaro, Italy

## Abstract

**Background:**

Farnesyltransferase inhibitors (FTIs) are anticancer agents developed to inhibit Ras oncoprotein activities. FTIs of different chemical structure act via a conserved mechanism in eukaryotic cells. They have low toxicity and are active on a wide range of tumors in cellular and animal models, independently of the Ras activation state. Their ultimate mechanism of action, however, remains undetermined. FTase has hundred of substrates in human cells, many of which play a pivotal role in either tumorigenesis or in pro-survival pathways. This lack of knowledge probably accounts for the failure of FTIs at clinical stage III for most of the malignancies treated, with the notable exception of haematological malignancies. Understanding which cellular pathways are the ultimate targets of FTIs in different tumor types and the basis of FTI resistance is required to improve the efficacy of FTIs in cancer treatment.

**Results:**

Here we used a yeast-based cellular assay to define the transcriptional changes consequent to FTI peptidomimetic administration in conditions that do not substantially change Ras membrane/cytosol distribution. Yeast and cancer cell lines were used to validate the results of the network analysis. The transcriptome of yeast cells treated with FTase inhibitor I was compared with that of untreated cells and with an isogenic strain genetically inhibited for FTase activity (*Δram1*). Cells treated with GGTI-298 were analyzed in a parallel study to validate the specificity of the FTI response. Network analysis, based on gene ontology criteria, identified a cell cycle gene cluster up-regulated by FTI treatment that has the Aurora A kinase IPL1 and the checkpoint protein MAD2 as hubs. Moreover, TORC1-S6K-downstream effectors were found to be down-regulated in yeast and mammalian FTI-treated cells. Notably only FTIs, but not genetic inhibition of FTase, elicited up-regulation of ABC/transporters.

**Conclusions:**

This work provides a view of how FTIs globally affect cell activity. It suggests that the chromosome segregation machinery and Aurora A association with the kinetochore as well as TORC1-S6K downstream effectors are among the ultimate targets affected by the transcriptional deregulation caused by FTI peptidomimetics. Moreover, it stresses the importance of monitoring the MDR response in patients treated with FTIs.

## Background

Farnesyl transferase (FTase) and Geranylgeranyl transferase I (GGTase I) are heterodimeric enzymes that catalyze the transfer of C-15 or C-20 lipid moieties, respectively, to the C-terminal cysteine of proteins having CAAX motifs at their C-terminus, the last amino acid discriminating among the two enzyme substrates [1]. The observation that Ras oncoproteins require farnesylation for membrane binding and malignant activity led to the development of drugs targeting FTase. As FTase structure and function has been conserved throughout evolution, the first farnesyl transferase inhibitor, Manumycin A, was selected using a yeast-based screening system [2]. Over the past decade, improved chemically-synthesized FTase and GGTase I inhibitors (FTI and GGTI, respectively) were tested in preclinical models. Surprisingly, they were active on a wide range of tumors independently of their Ras oncogenic status. Nowadays, it is clear that there are hundreds of FTase substrates and the wide spectrum of action of FTIs arises from the large number of farnesylated proteins acting at different levels in oncogenic pathways and pro-survival pathways [3].

Although preclinical studies showed that FTIs induce tumor growth inhibition rather than tumor regression and their ultimate targets in cells remained largely undefined, they entered clinical trials. Promisingly, FTIs synergize with taxanes and show some efficacy in the treatment of breast cancer when administrated in combinatorial therapy. Unfortunately, when administrated as a single agent, they failed clinical trials at stage III for most of the malignancies tested, with the notable exception of haematological malignancies. This failure has been largely attributed to the scarce knowledge of how FTIs ultimately act in the cell [4-9].

Collectively, clinical and preclinical studies have shown that FTIs are anti-proliferative agents that have low toxicity for normal cells. Moreover, the efficacy of structurally diverse FTI molecules is highly consistent in different organisms, showing that they act via a conserved mechanism in eukaryotic cells [5,8]. Proteomic approaches aimed at identifying proteins differentially prenylated upon FTI treatment have shown that, in addition to Ras oncoproteins, DNAJ, laminin A, nucleosome assembly protein NAP-1, peroxisomal biogenesis factor 1 and annexin are differentially prenylated using FTIs at clinically relevant dosages [10]. More classical biochemical approaches related FTI anti-proliferative action to defects in the attachment of farnesylated proteins, such as CENP-E and CENP-F, to kinetochores [11-13] or poor prenylation of RheB [3]. Unfortunately, the correct minimal balance of farnesylated and non-farnesylated proteins within cells is often unknown. Taken together, these approaches have been unable so far to clearly correlate the FTI antiproliferative action with the prenylation status of a given protein basically due to: i) the large number of farnesylated proteins in human cells; ii) their multiple roles in different processes leading to proliferation and, last but not least, iii) the difficulty in correlating the lack of prenylation of an FTase target with the FTI antiproliferative action [4,14].

To predict FTI efficacy at the clinical level it is necessary to devise novel genomic strategies to further decipher how FTIs ultimately affect cellular activity. Which off-targets they might affect and how FTI resistance is achieved are major challenges for today's studies. Promising results towards the goal of predicting FTI efficacy in clinical practice were recently reported by profiling the expression signatures of newly diagnosed Acute myelogenous leukemia (AML) patients treated with TIPIFARNIB (Zarnestra). The ratio of expression levels of two genes, *RASGRP *and *APTX*, appears to be predictive of the Tipifarnib response in AML patients, although it remains unclear how this might operate [3,8,15].

From the above it is clear that a better knowledge of FTI action at the cellular level is required. Here we have used a yeast genetics strategy and FTI peptidomimetics administrated at dosages that do not appreciably affect Ras cytosolic/membrane distribution to identify the network of proteins transcriptionally deregulated by this class of drug. Network analysis and follow-up studies were performed in yeast and in mammalian cancer cell lines using biochemical approaches and high-throughput high-content image analysis. Collectively, the results indicate that the ultimate peptidomimetic FTI targets are proteins in the nucleus acting at the kinetochore having as hubs the Aurora A and MAD2 proteins. We show that Aurora A is indeed mislocalised in HeLa cells upon treatement with the FTI peptidomimetic FTI-277. Furthermore, we show that proteins acting at the crossroads of the Ras/PKA and TORC/S6K pathways are down-regulated in yeast genetically impaired in FTase activity or treated with an FTI peptidomimetic. Consistent with this, HeLa and MCF-7 cells show poor phosphorylation of the ribosomal S6 protein, a target of TORC/S6K during starvation. Moreover, we show that a FTI peptidomimetic up-regulated the MDR gene response.

## Results and Discussion

### Basic assumptions and set-up of the assay conditions

It is well established that the basic components of the prenylation, transcriptional, cell cycle and trafficking machineries are conserved between yeast and humans. Moreover, *S. cerevisiae *has proven to be a powerful genetic tool to elucidate the mode of action of clinically relevant compounds at the genomic level [16-19]. We reasoned that if there is a basic common mechanism by which FTIs act as antitumor agents in eukaryotic cells [3,5,8,20] then this mechanism could be identified in yeast cells based solely on FTase activity inhibition and the results reciprocated in mammalian cells, irrespective of the FTI compound used in the two organisms.

Human and yeast FTases are conserved in structure and function. They are constituted by two subunits, called RAM1 and RAM2 in yeast cells. The β subunit RAM2 is shared with GGTase I, while the *alpha *subunit is specific for each enzyme, RAM1 for FTase and CDC43 for GGTase I [1]. Importantly, for the aims of this study, cells deleted for the *RAM1 *gene (*Δram1*) are viable as essential proteins, that cannot be farnesylated in the absence of FTase activity, are geranylgeranylated by GGTase I. Thus, budding yeast provides a unique genetic tool to decipher the global response to FTIs and to prove that FTI antiproliferative action relies solely on the inhibition of FTase activity.

To define the expression signature of FTI- or GGTI-treated yeast cells we chose well-studied and commercially available compounds. All of them had been already tested for their antiproliferative action in cancer cell lines. To be suitable for our study, we applied the following constraints. The compound should: 1) be cell permeable (to be administrated in the growth media); 2) be already demonstrated to possess a higher specificity either for FTase or GGTase I in a mammalian cellular system; 3) be active, as an anti-proliferative drug, at the same concentration in yeast and mammalian cells. Moreover, we were interested to study the effects on transcription independently of Ras. Thus we searched for an FTI concentration that could elicit an antiproliferative action in yeast and in mammalian cells without substantially affecting Ras membrane/cytosolic distribution.

Based on the above criteria, the commercially available FTIs we tested were the FTase inhibitor I (N-[2(S)-[2(R)-Amino-3-mercaptopropylamino]-3-methylbutyl]-Phe-Met-OH) and FTI-277 (Merck-Calbiochem) and as a GGTase inhibitor GGTI-298 (Merck-Calbiochem). According to the manufactures, FTase inhibitor I and FTI-277 are peptidomimetics that exert an antiproliferative action in human tumor cell lines at concentrations above 21 nM. In experimental work these compounds are generally used at a concentration range between 0.5-10 μM [21-24].

To set up the appropriate cell assay conditions that fulfill the above-mentioned criteria, we tested FTase inhibitor I and FTI-277 in parallel with the antibiotic Manumicyn A (Merk-Calbiochem). Manumycin A, an FTI that was selected in yeast cells, has poor specificity for either FTase or GGTase I [2].

**Figure 1:**
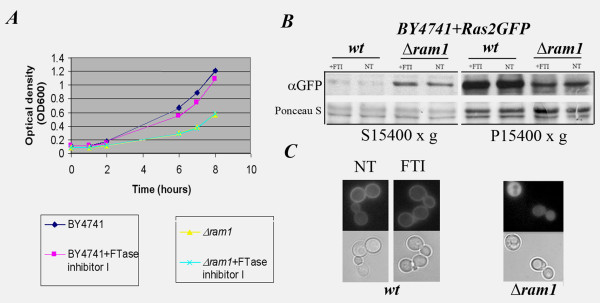
**Effect of FTase inhibitor I on growth and Ras2 localization in wt and *Δram1 *cells**. **A**, BY4741 but not *Δram1 *cells are sensitive to 10 μM FTase inhibitor I. Growth curves were performed in parallel cultures with or without 10 μM FTase inhibitor I. Optical density (OD_600_) measurements were taken at the indicated time points. **B**, GFP-Ras2 localization in FTI-treated BY4741 and *Δram1 *cells. Total cell extracts were clarified at 3500 × g and fractionated into cytosolic (S15400 × *g*) and membrane (P15400 × g) fractions as previously described [50]. 30 μg of proteins were separated by SDS-PAGE and immunoblot analysis was performed using α-GFP (upper panels) after Ponceau S staining (lower panels) of nitrocellulose membranes. **C**, Fluorescence (upper panels) and DIC (lower panels) images of cells used in **(B) **prior to fractionation.

Summarising a large number of growth tests that were performed with different concentrations ranging from 1 to 30 μM of FTI-277, FTase inhibitor I and Manumycin A in liquid synthetic complete media (SCD) or rich liquid media (YPD) containing 2% glucose, we found that 10 μM Manumycin A was sufficient to reduce the doubling time of yeast cells with different genetic backgrounds (Table 1: K699, W303 and BY4741) in SCD. The duplication time of the BY4741 strain is significantly reduced in the presence of 10 μM Manumycin A or 10 μM FTase inhibitor I, but is not affected by the use of similar dosages of FTI-277 or GGTI-298 (Fig. 1A and data not shown). As expected, *Δram1 *BY4741 cells, lacking FTase activity due to deletion of the *RAM1 *gene, are not affected by a 10 μM concentration of FTIs (Figure 1A) while Manumycin A shows some toxicity in this strain as it also targets GGTase I (data not shown). The strain K699, a derivative of strain W303, showed some degree of sensitivity with an average duplication time of 84 min in the absence of FTase inhibitor I and 98 min with 10 μM FTase inhibitor I, 119 min in the presence of Manumicyn A, but was not affected by FTI-277 or GGTI-298.

**Table 1:** Strain list

Strains	Genotype
W303-1B	MATα leu2, ura3, his3, TRP1, ade2, can^R ^/wt mit+

BY4741	MATa, leu2, ura3, his3, met15

YDL090C	MATa, leu2, ura3, his3, met15, Δram1:Kan^r^

K699	MATa, ade2-1 trp1-1 can1-100 leu2-3,112 his3-11,15 ura3 GAL psi+

In summary, all strain backgrounds tested are sensitive to 10 μM Manumycin A in SCD liquid media. The average replication time during the exponential phase of growth of strains BY4741 and K699 is slower in the presence of 10 μM FTase inhibitor I. No effect on the replication of any tested yeast strain was observed upon addition of 10 μM FTI-277 or GGTI-298. Based on these tests we chose 10 μM FTase inhibitor I to add to liquid media and the yeast strain BY4741 and its isogenic *Δram1 *derivative (EUROSCARF) for this study.

To determine if FTase inhibitor I alters Ras membrane binding, BY4741 and Δ*ram1 *cells expressing GFP-Ras2p were treated or not with 10 μM FTase inhibitor I in parallel experiments. The amount of GFP-Ras2p bound to intracellular membranes or in the cytosol was examined by immunoblot analysis, using an anti-GFP antibody, of cell extracts fractionated by differential centrifugation. A marked reduction in the amount of GFP-Ras2p bound to intracellular membranes was clearly observed in Δ*ram1 *cells compared to untreated BY4741 cells (Figure 1B, panel αGFP P15400 × g). FTI treatment did not change the amounts of GFP-Ras2p bound to intracellular membranes either in BY4741 or Δ*ram1 *cells (Figure 1B, panel αGFP). Fluorescence microscopy analysis of the cells prior to fractionation corroborated these data (Figure 1C).

Thus, 10 μM of the FTase inhibitor I peptidomimetic: a) reduces the duplication time during exponential growth of BY4741 but not *Δram1 *cells; 2) does not substantially alter the membrane/cytosol distribution of the Ras2 protein. Unless otherwise specified, in the remaining part of the paper the use of "FTI treatment" refers to the use of 10 μM FTase inhibitor I.

### The chromosome segregation and the Multidrug-Resistance machineries are up-regulated during FTI treatment

The effects of FTI on yeast global gene expression were determined using commercially available dEST microarrays (Yeast 6.4K Array; UHN microarray Center, Toronto Canada). Briefly, these are glass arrays double-spotted with 6,240 unique yeast ORFs and 160 gene controls of *Arabidopsis thaliana*. The information on the spotting and experimental design of the Yeast 6.4K Arrays are available at the manufacture's web site http://www.microarray.ca/, detailed in the Methods section and in the Additional file 1. We performed at least four biological replicates per sample. Cy3/Cy5 dye-swapping method was used to differentially label fluorescent cDNAs obtained by reverse mRNA transcription of FTase inhibitor-treated and untreated cells. After cDNA labelling, mixing and hybridization the arrays were scanned and normalised using GenePix6 software tools (Molecular Devices). Lowess normalization was performed by using the Acuity software (Molecular Devices). Genes flagged absent (too low intensity over background) or flagged bad were excluded from the calculations. The expression levels of all genes present in each data set was visualised using Gene@work (IBM research; additional file 1 and 2: Figure S1, S2 and S3). Comparing each color panel (left panels in additional file 2 Figure: S1, S2 and S3, respectively) with the mean expression color panel calculated for each data set (right panel in additional figure S1, S2 and S3, respectively), it is possible to observe an overall good reproducibility: up-regulated and repressed genes cluster at the two opposite sides of the color panels in each biological replicate.

T-test of Acuity program (Molecular Devices) was used to statistically filter the normalised data and to obtain the statistically significant hits. Quantitative Real-time PCR was finally used to validate ten hits, randomly chosen among the up-regulated and down-regulated genes of FTI treated samples (Additional file 2: Figure S4 and Table S1).

To obtain information about the functional relationships of the statistically significant hits, they were further analysed using the Gene Ontology criteria at the *S. cerevisiae *Gene Ontology (SGO) annotation database (http://www.yeastgenome.org/GOContents.shtml, update November 2009). Gene clusters sharing a similar ontology category were further dissected for their network relationship using the STRING database and related tools at http://string.embl.de (*STRING*- *Known and Predicted Protein-Protein Interactions*, version 8.2; highest confidence search score set 0.900, no text mining).

Using a T-test confidence score set at 80%, a total of 69 transcripts were classified as up-regulated and 35 down-regulated at least 0.5 fold upon FTI treatment (Additional file 2: Table S2).

**Figure 2:**
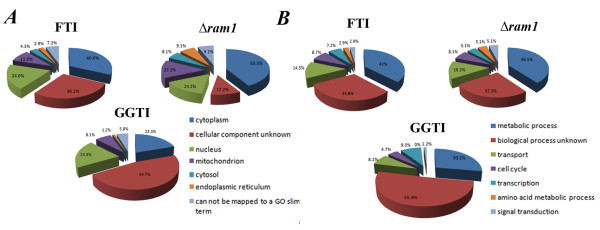
**Comparative gene ontology analysis**. Pie representation of up-regulated genes using the gene ontology GO-Slim tool at the *Saccharomyces *Gene Ontology Data base binned for **(A) **compartment of action and **(B) **biological processes. FTI, BY4741 cells treated with 10 μM FTase inhibitor I; GGTI, BY4741 cells treated with 10 μM GGTI298; *Δram1*, untreated YDL090C cells compared to untreated BY4741 cells.

Binning the 69 up-regulated transcripts for compartment of action, using **Super GO-Slim Component **at the SGO database (Figure 2A, panel FTI), showed that 24.6% of them encode proteins that reside within the nucleus (Additional file 2: Table S3). Of these, a statistically relevant (p < 0.01) group has a function in chromosome segregation (namely, *AME1*, *FIN1*, *SCC2*, *CNN1*, *NNF1*) and/or in microtubule organization (namely, *DAD1*, *FIN1*, *DUO1*; Additional file 2: Table S4). *AME1*, *CNN1*, *DAD1*, *DUO1 *and *NNF1 *share a function at the kinetochore (Additional file 2: Table S5).

**Figure 3:**
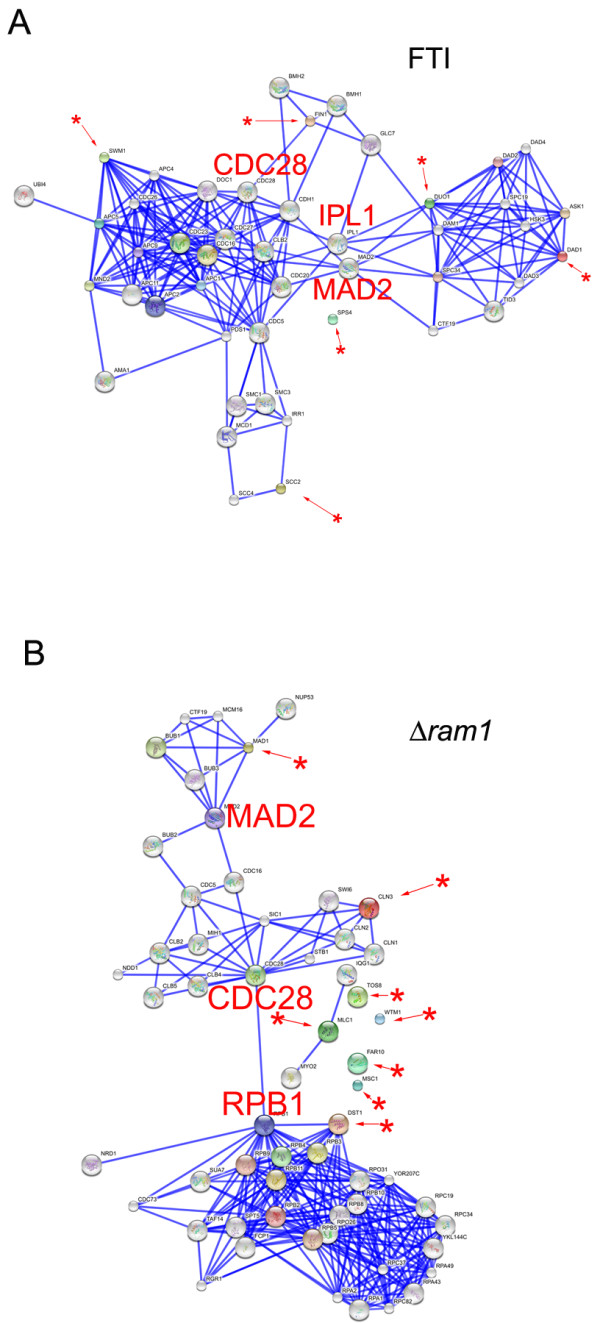
**Network analysis of the cell cycle up-regulated transcripts in FTI-treated BY4741 and in *Δram1 *cells**. Confidence view image of gene clusters acting in the cell cycle and up-regulated in **(A) **10 μM FTI treated BY4741 cells and **(B) ***Δram1 *cells using the *STRING *analysis tool. Analyses were performed by setting the confidence score at the highest confidence (score = 0.900) and excluding text mining. Asterisks with a red arrow indicate the input proteins. The position of nodes CDC28, MAD2, IPL1 and RPB1 within the network is highlighted.

The possible relationship among the genes binned in the cell cycle group according to GO criteria (*DAD1*, *DUO1*, *FIN1*, *SCC2*, *SPS4*, *SWM1*; Additional file 2: Table S6) were further dissected using STRING tools (see above). With the exception of SPS4, the aurora kinase IPL1 and the checkpoint protein MAD2 were identified as possible hubs of this group (Figure 3A). The serine-threonine kinase Aurora A controls various mitotic events including chromosome segregation in yeast as well as in human cells. The aurora A mRNA varies throughout the cell cycle and peaks during G_2_/M. MAD2 is a spindle-assembly checkpoint complex component. Its activity is required to delay the onset of anaphase in cells with defects in mitotic spindle assembly [25].

Thus, the clustering and network analyses of the cell cycle group up-regulated by FTI treatment suggest that proteins acting at the kinetochore and at the spindle-assembly checkpoint complex are among the final targets of FTI peptidomimetics and point to the Aurora A kinase IPL1 and the MAD2 protein at the hubs of this transcriptional response (Figure 3A). See below for validation of this assumption in mammalian cells.

Binning of the 69 up-regulated genes by biological process using **Super GO-Slim Process **tool (Figure 2B and Additional file 2: Table S6) identifies genes regulating the transcription of plasma membrane (PM) transporters as responding to FTI treatment.

The up-regulation of *PDR1 *and the sugar transporters *HXT16 *and *HXT17 *(Additional file 2: Table S6) is of note in this group, as there is a relationship between *HXT *genes, ABC transporter up-regulation and drug resistance in yeast as well as in mammals. PDR1 is the major regulator of the expression of the PM transporters known as ATP-binding cassettes (ABC) such as *PDR5 *and *PDR10*. ABC transporters are P-glycoproteins (Pgp) that represent the yeast counterparts of the human multidrug-resistance proteins (MDRs). ABC transporters and Pgp are the major contributors to multidrug resistance by regulating drug efflux of several unrelated compounds [26,27]. The human Pgp MDR1 functions as an efflux pump for different anticancer agents such as anthracyclines, Vinca alkaloids, and taxanes [28]. The yeast hexose transporters (HXT) are homologues of the mammalian glucose transporters (GLUT). GLUT-1 is consistently up-regulated in cells with K-RAS or B-RAF mutations in colorectal cancer cell lines [29] and is responsible of enhanced glucose uptake, glycolysis and increased survival in low-glucose conditions in these cells. In yeast, moreover, PDR1 regulates the hexose transporters *HXT9 *and *HXT11 *[30,31]. Notably, overexpression of these latter hexose transporters increases sensitivity to drugs [31,32]. In summary, given these correlations between *MDR *and *HXT *genes and considering the network analysis results, it is conceivable to think that the up-regulation of *PDR1 *and *HXT *genes in FTI-treated cells is part of the same cellular circuit, controlling the ability of the cells to expel drugs via MDR activation.

**Figure 4:**
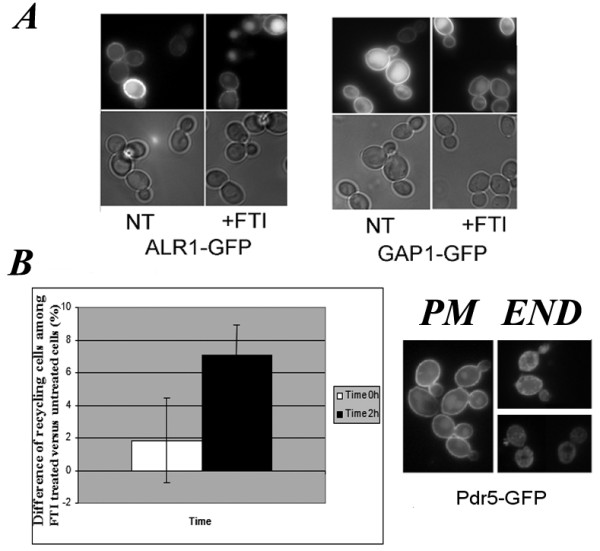
**Multidrug transporter GFP-Pdr5 recycling is affected by FTI treatment of wt cells**. **A**, fluorescence (upper panels) and DIC (lower panels) images of K699 cells expressing Alr1-GFP or Gap1-GFP proteins. **B**, Representative fluorescence images of cells expressing PDR5-GFP with plasma membrane (PM) or endosomal (END) localization are shown in the right panels. Images were taken in a blind fashion at time 0 h (T0) or 2 h (T2 h) after 10 μM FTase inhibitor addition and at least 100 cells were counted per sample. The graph on the left represents the increase (%) of END-type over PM-type cells observed in FTI-treated versus untreated cells. The values are the mean of 3 independent experiments. Bar indicates standard deviation. 10 μM FTase inhibitor I (+FTI); untreated (NT).

To confirm that FTI treatment activates a multidrug resistance response also at the protein level, we examined the localization of the ABC transporter PDR5, which is transcriptionally regulated by PDR1, in treated and untreated yeast cells. For this we used a GFP-tagged PDR5 (GFP-PDR5) protein and as controls the general amino acid permease GAP1 (*YKR039W*; GFP-GAP1) and the metal ion and magnesium transporter ALR1 (*YOL130W*; GFP-ALR1) by epifluorescence microscopy in time-lapse experiments (Figure 4A, respective panels). GFP-PDR5 localizes mainly at the plasma membrane (Figure 4B, panel PM; [33]) during exponential growth while it accumulates in endosomal and vacuolar compartments when cells reach stationary phase [33].

The localization of GFP-PDR5, GFP-ALR1 and GFP-GAP1 was scored at different time points prior to and after drug addition during the exponential growth phase. No changes in localization of GFP-ALR1 or GFP-GAP1 were observed 2 h after the addition of FTase inhibitor I (Figure 4A, respective panels). Within 2 h of FTase inhibitor I addition we observed a slight increase in the number of cells with GFP-PDR5 in endosomal structures (Figure 4B) that further increases when cells are left without agitation for a further 30 min. We conclude that FTase inhibitor I alters PDR5 recycling from the plasma membrane. We cannot rule out the possibility that an increase in the amount of PDR5 due to PDR1 up-regulation after FTI treatment might cause an "GFP-PDR5 endosomal jam" in FTI-treated cells.

To further corroborate the specific effect of FTI treatment on ABC transporter expression/recycling over other pathways, we scored several GFP (or RFP)-tagged PM proteins as well as proteins involved in intracellular trafficking of lipids and proteins, in vesicle and organelle motility and/or cytokinesis in FTI-treated and untreated cells (see list of constructs in Table 2). With the exception of PDR5, no differences in localization between FTI-treated and untreated cells were observed for any of the proteins listed in Table 2.

**Table 2:** Plasmids list

Plasmid construct	Marker	Replication Origin	Reference
Myo1-GFP pRS316	URA	CEN	E. Bi

Cdc3-GFP pRS316	URA	CEN	G. Ammerer

Mrs6-GFP YEp352	URA	2 μ	[53]

Fba1-GFP pUG35	URA	CEN	S. Wacha

Ras2-GFP pUG34	HIS	CEN	This work

Sec2-GFP pUG35	URA	CEN	[50]

Sec4-GFP pUG36	URA	CEN	[50]

Mlc1-GFP pUG36	URA	CEN	[50]

Alr1-GFP pUG34	HIS	CEN	A. Graschopf

Pdr5-GFP pRE104	HIS	CEN	[32]

Gap1-GFP pTPQ99	URA	CEN	C. Walch-Solimena

Sec7-RFP pTPQ127	LEU	CEN	C. Walch-Solimena

This result confirmed that FTI treatment alters the MDR response in yeast cells at the transcriptional as well as at the protein level.

### FTI down-regulates transcripts of key components of the TORC1-S6K pathways

Thirty-five genes were found to be down-regulated in FTase inhibitor I-treated cells (Additional file 2: Table S2). Among them, 8.6% share a function in the cell cycle (*FAR7*, *RIM15*, *ZIP2*), and a similar percentage in transcription (*HAP5*, *MET18*, *RPA135*; Table S7).

Network analysis of the cell cycle-associated RIM15, FAR7 and ZIP2 gene cluster using the STRING software identified the cyclin CLN2 as a hub of this protein network (Additional file 2: Figure S5). The G1 cyclin CLN2, by activating the cyclin-dependent kinase Cdc28p, is critical for re-entry into the mitotic cell cycle after G_0 _arrest and for G1/S progression during vegetative growth and after pheromone arrest. Chemically different FTIs were reported previously to cause such a defect in mammalian cells [3,5].

The finding of *RIM15 *in the group of down-regulated cell-cycle associated genes suggests an action of FTI on the ability of cells to respond to nutrient starvation. RIM15 is a distinct member of the PAS kinase family regulating cell growth and division in response to RAS/PKA and/or TOR/SCH9 signalling networks, through the transcription factors Msn2/4 and Gis1 (Additional file 2: Figure S5; [34]). In particular, RIM15 positively regulates proper entry into stationary phase under nutrient starvation [34,35]. STRING network analysis of the RIM15, FAR7 and ZIP2 gene cluster (Additional file 2: Figure S5) shows a direct link between RIM15 and the TORC1-responsive kinase SCH9. The Sch9 kinase is the yeast orthologue of the mTORC1 substrate S6K1 [36]. Similarly to the mTORC1-S6K1 signalling pathway, it plays an essential role in regulating life-span in response to nutritional and environmental stress cues [36,37]. In human tumors, hyperactivation of mTORC1, via the S6K pathways, leads to the hyperphosphorylation of ribosomal S6 protein (RPS6; [38]). The evolutionary conservation of the pathway controlled by TORC1 and S6K1 [36] and of the FTI mode-of-action, prompted us to test if FTI peptidomimetics down-regulate TORC1-S6K1 signaling under starvation conditions in HeLa and MCF-7 cells.

Thus, to corroborate the finding that the RIM15 kinase is down-regulated in yeast cells, and consequently the response to nutrient starvation altered by FTI treatment, we investigated the level of RPS6 phosphorylation in HeLa and MCF-7 cells treated with 5 μM FTI-277. We choose this compound and concentration here essentially for three reasons: i) most of the work performed with FTI peptidomimetics in mammalian cells were performed with this compound; ii) when we started this particular study FTI inhibitor I was no longer commercially available; iii) 5 μM FTI-277 was previously reported to exert an antiproliferative action on HeLa and MCF-7 cells [3,4,8].

**Figure 5:**
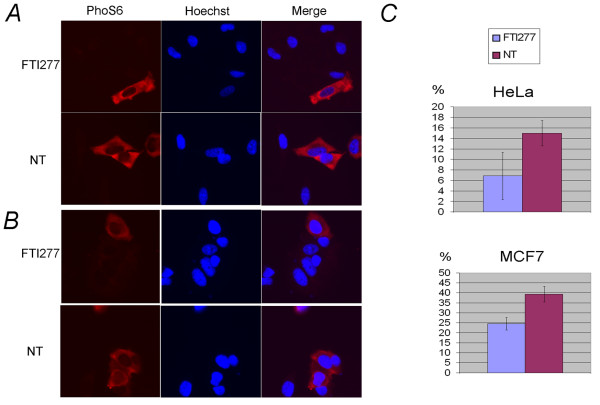
**FTI-277 treatment reduces the phosphorylation of ribosomal protein S6 in HeLa and MCF-7 cells**. The levels of RPS6 (p70) phosphorylation in HeLa and MCF-7 cells were measured using the ScanR analysis software based on the intensity of AlexaFluor 555-conjugated PhoS6 antibody (Ser 235/236: Cell Signalling) recorded from images acquired from samples treated with 5 μM FTI-277 (panel FTI-277) or not treated (panel NT) in the presence of 0.1% serum for 24 h. Five wells per condition were considered. Cells were seeded in a 96-well plate and fixed and stained as described in Methods. Sixteen images were acquired randomly per well with a 20× objective using the RFP filter. The mean intensity within the red channel was automatically calculated by ScanR analysis software considering at least 600 cells. **A**, Images representative of FTI-277-treated and untreated HeLa cells stained with PhoS6 antibody and Hoechst, single and merged pictures are shown. **B**, Images representative of FTI-277-treated and untreated (NT) MCF-7 cells, stained as in **(A)**; **C**, Analysis of the distribution of the phosphorylated RPS6 population in HeLa and MCF-7 cell lines. Hoechst staining was used for cell identification (main mask), as described in Methods. The minimal threshold of the PhoS6 signal was set at mean intensity 43.8 for HeLa and at 63.8 for MCF-7 cells based on the background intensity. The percentage of cells having a PhoS6 signal localised within this gate was calculated and plotted in the graph.

The state of RPS6 phosphorylation (PhoS6) in FTI-277 treated (FTI277) and untreated (NT) cells during serum starvation was determined by automated high-throughput fluorescence microscopy using the ScanR microscopy station (Olympus) for acquisition and multiparametric analysis of images (HT/HCA; Figure 5). For this, cells were seeded in five-well replicates in a 96-well plate. The cells were left to adhere for 24 hours prior to the addition of 5 μM FTI-277 under starvation conditions as previously described by [39]. After 48 hours of treatment, sixteen images were acquired randomly from each sample using the RFP filter cube (U-MRFPHQ Olympus) to detect the anti-PhoS6 antibody conjugated to AlexaFluor555 and using the DAPI filter (Olympus) for Hoechst-stained nuclear DNA (Figure 5A and 5B). Cells were counted and cell cycle distribution established based on the Hoechst mean intensity and total intensity signal/area. Then, RPS6 phosphorylation levels were evaluated based on the mean intensity values of treated compared to untreated samples in the RFP channel. Overall, at least 600 cells per condition were counted and the PhoS6 levels were analyzed on the whole population using a mask containing both cytosol and nuclei, or calculated only considering the fluorescence intensity within the main mask (nuclear area).

We observed that FTI-277 reduced the overall PhoS6 signal, corresponding to phosphorylated RPS6, by 23.6% in HeLa cells and by 28.8% in MCF-7 cells compared to untreated cells. A closer inspection of the RPS6 distribution with the area containing the highest PhoS6 signal reveals that FTI-277 treatment reduced the number of cells present in this gate by 54% in HeLa cells and by 37.6% in MCF-7 cells compared to the respective untreated samples (Figure 5C respective panels).

All together, these analyses show that in yeast an FTI peptidomimetic down-regulates the transcription of genes acting at the cross-roads of the RAS/PKA and TORC1-SK6 pathways regulating ribosomal protein expression and cell cycle progression in response to nutritional stress and starvation. This response is conserved in mammalian cells [36]. Indeed, our data show that the TORC1-SK6-dependent phosphorylation of RPS6 (p70) is strongly down-regulated by FTI-277 during starvation.

### Transcriptional deregulation of TOR-responding genes as well as of central components of the MAP-kinase pathway and tumor suppressor genes are features of *Δram1 *cells

We analysed the expression profile of Δ*ram1 *cells and compared it to that of the isogenic BY4741 cells and to FTI-treated cells to highlight the effects of chronic versus acute inhibition of FTase. As previously mentioned, *Δram1 *cells deleted for the gene encoding the RAM1 subunit of the FTase enzyme completely lack FTase activity.

Cell growth, RNA extraction and array preparation and analysis were performed in parallel with those reported above to minimise experimental differences such as media, growth conditions, methods of RNA extraction or cDNA labelling and the microchip batch.

Ninety-nine and thirty-eight transcripts were identified to be up-regulated or down-regulated, respectively, at least 0.5 fold in Δ*ram1 *cells compared to BY4741 cells (Additional file 2: Table S8; T-Test set 80% confidence). Consistent with the fact that GGTase I prenylates FTase substrates in *Δram1 *cells [1], up-regulation of *CDC43/CAL1*, encoding the beta subunit of the GGTase I, was observed in *Δram1 *cells.

Clustering the up-regulated hits for compartment of action, using **Super GO-Slim Component **(Additional file 2: Table S9), or for biological process (Additional file 2: Table S10), using **Super GO-Slim Process **at the SGO database, shows that the frequency of gene clusters falling in the same compartment of action or sharing a function in the same biological process is similar between *Δram1 *and FTI-treated cells (Figure 2A: Compartment of action and Figure 2B: biological processes).

Unlike the 10 μM FTase inhibitor I-treated cells, no statistically significant group (p-value < 0.01) sharing a similar function within the nucleus was identified. Binning the 99 up-regulated hits in *Δram1 *cells for biological process shows that 5.1% of the transcripts encode proteins acting in signal transduction (*CDC43*, *GPG1*, *SDP1*, *TEP1*, *TPK1*; Figure 2B and Additional file 2: Table S10). We note that only 2.9% of the genes fall into this category in the case of FTI-treated cells (Additional file 2: Table S6).

Within the signal transduction group (Additional file 2: Table S10) of up-regulated genes in *Δram1 *cells were the catalytic subunit of PKA (the cAMP-dependent protein kinase type 1, *TPK1*), the protein-tyrosine phosphatase *SDP1 *and the yeast orthologue of the human tumor suppressor gene *PTEN *(the *TEP1 *gene).

Sdp1p, a stress-inducible dual-specificity MAP kinase phosphatase is induced by several stress conditions in an Msn2p/Msn4p-dependent manner [40], which negatively regulates the Slt2/MPK1 serine/threonine mitogen-activated kinase by direct dephosphorylation. In yeast, the Slt2p/MPK1 kinase is part of a signaling cascade required to maintain cell wall integrity and progression through the cell cycle acting downstream of PKC pathways [41]. Sdp1p is also capable of dephosphorylating the closely related mammalian MAPK ERK2 [42]. The yeast counterpart of the tumor suppressor gene *PTEN *(phosphatase and tensin homologue deleted on chromosome 10), tensin-like phosphatase *TEP1*, was also identified. In mammals *PTEN *is either deleted or inactivated in a very high percentage of breast, endometrial, brain, and prostate cancers. Homozygous deletion of *PTEN *in mice is embryonic lethal and mice with only one functional copy of the gene are more likely to develop tumors [43]. Thus, chemical and genetic FTase inhibition elicits both common and non-overlapping transcriptional responses. The common set of responses involves genes in the TORC1-S6K1 signalling pathway, but differently from the FTIs, key components of Ras/PKA pathways are affected by *RAM1 *deletion. The chronic versus acute FTase inhibition response reflects the fact that we set the FTI concentration at a level that does not affect Ras pathways while this pathway is necessarily altered in *Δram1 *cells.

Within the 5.1% of transcripts up-regulated and falling within the transcription category (*AFT1*, *CEG1*, *DST1*, *SOH1*, *TOS8*) there are mainly RNA polymerase II transcriptional regulators (Additional file 2: Table S10), while in FTI-treated cells this category comprised MDR gene transcription regulators.

Similarly to the case of FTI-treated cells, 8.1% of genes up-regulated in *Δram1 *cells fall into the cell cycle category (Figure 2B*Δram1 *and Additional file 2: Table S10, cell cycle) and network analysis by STRING (Figure 3B) identified CLN3, DST1 and MAD1 as forming a network having nodes at the catalytic subunit of the cyclin-dependent kinase CDC28, the spindle checkpoint protein MAD2 and the RNA polymerase II subunit RPB1. MAD1 (Mitotic arrest deficient protein 1) is a central component of the spindle assembly checkpoint, thought to recruit MAD2 to unattached kinetochores.

Among the 38 down-regulated genes (Additional file 2: Table S8, down-regulated) in Δ*ram1 *cells acting in the cell cycle and signal transduction (Additional file 2: Table S11) were the mitogen-activated protein (MAP) kinase kinase kinase BCK1, which acts in the protein kinase C signaling pathway, the cytoskeletal motor DYN1, required in anaphase for mitotic spindle elongation, and SAC7, the GTPase activating protein (GAP) for Rho1p.

Sac7p acts as a negative regulator of the geranylgeranylated small GTPase RHO1 that plays an essential role in signaling to the actin cytoskeleton. Moreover, Sac7p is linked to the cell cycle, being a potential Cdc28p substrate. Interestingly, deletion of *SAC7 (Δsac7*) suppresses *tor2 *mutations and increases MPK1 activation [44]. Thus, one might expect that the constitutive down-regulation of *SAC7*, observed in *Δram1 *cells, must result in MPK1 hyperactivation. That this is the case is supported by the above-mentioned up-regulation of Sdp1p. Clearly, it will be the balance between these two opposite activities on the MPK1 protein that will finally regulate *Δram1 *cell proliferation and stress responses. The human tumor suppressor gene *DLC-1 *is a Sac7 family member. DLC-1 is normally expressed in many adult human tissues, and is down-regulated or absent in a number of common human cancers, including breast, colon, ovarian, uterine, gastric, lung, pancreatic, prostate, renal and nasopharyngeal tumors [45].

### GGTI-298 treatment deregulates genes of unknown function

The specificity of the response to FTIs was validated by analysing the transcriptional signatures of BY4741 cells treated or not with 10 μM GGTI-298.

As mentioned above, GGTI-298 is a cell permeable compound prevalently targeting GGTase I. Although no effect on the growth of yeast cells was observed in liquid or solid medium at this concentration, this does not necessarily mean that yeast are insensitive to this compound in our assay conditions.

After nomalization and statistical filtering with lowess and setting the T-test at 80%, 86 genes were found to be up-regulated in GGTI-298-treated yeast cells (Additional file 2: Table S12).

Binning these genes for compartment of action (Figure 2A panel GGTI and Additional file 2: Table S13) or for biological process (Figure 2B panel GGTI and Additional file 2: Table S14) showed that the overall transcriptional pattern differed markedly from that obtained by chemical or genetic inhibition of FTase activity. The most striking outcome from these analyses was that the majority of genes up-regulated by GGTI-298 have an unknown localization.

In particular, we note that of the 86 up-regulated genes only 4.7% are involved in the cell cycle (Figure 2B and Additional file 2: Table S14), compared to 8.7% and 8.1%, in FTI and *Δram1 *cells, respectively. Binning the up-regulated hits localised within the nucleus (Additional file 2: Table S15) for biological process using GoTerm Process tool at SGD showed that a significant (p-value cutoff < 0.01) group of genes act in RNA metabolic processes. Similar criteria applied to *Δram1 *cells gave no hits while a significant group of proteins acting at the kinetochore was observed in the case of FTase inhibitor 1.

The 95 genes showing statistically significant down-regulation in GGTI-298 treated cells (Additional file 2: Table S12) were binned by compartment of action, (Additional file 2: Table S16), and then classified by biological process, using GO Term Finder at SGD, producing a list of proteins that reside in the nucleus and are involved in rRNA maturation (Additional file 2: Table S17). A group functioning within the endoplasmic reticulum was also identified that act in lipid metabolism (Additional file 2: Table S18). It is also worth noting the down-regulation of *CDC43*, a subunit of GGTase-I, among the genes down-regulated in GGTI-298 treated cells confirming that GGTI-298 affects GGTase I function not only by inhibiting enzyme activity but also by down-regulating gene expression.

In conclusion, changes in the regulation of the yeast transcriptome in GGTI-298 treated cells do not correlate with those observed in FTase down-regulated cells.

### Effects of FTI-277 treatment on Aurora A kinase localization in HeLa cells

Network analysis of FTase inhibitor I-treated cells suggested Aurora Kinase IPL1 as one of the hubs of the cell cycle gene transcript network. Entry into mitosis is a highly regulated process promoted by the activated Cyclin B1/Cdk1 complex. Activation of this complex is partially dependent on the protein kinase Aurora-A. The phosphatase PPA2 promotes Aurora A degradation by dephosphorylating serine 51. The yeast Aurora A homologue Ipl1p was originally identified through mutations leading to increased chromosome mis-segregation [46].

**Figure 6:**
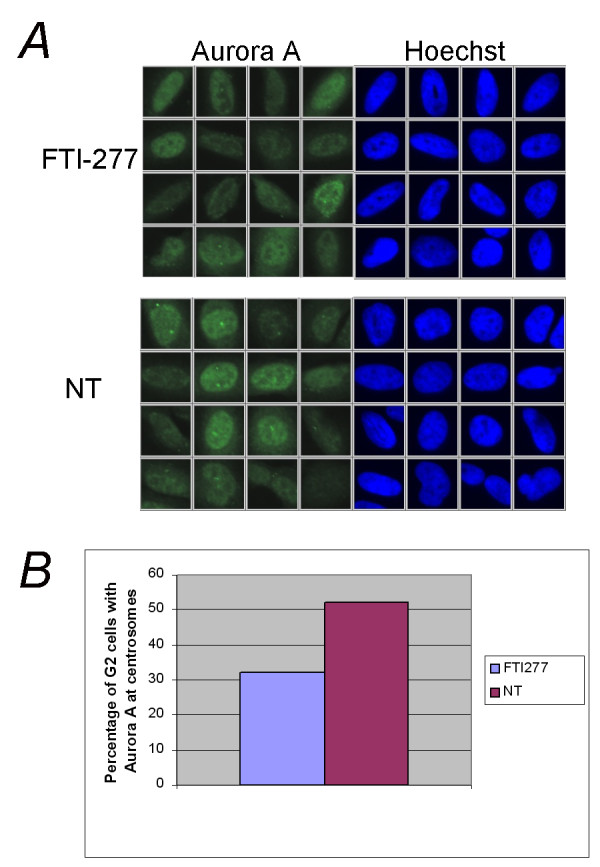
**FTI treatment impairs the localization of Aurora A in G2-M cells**. HeLa cells were seeded in 10 wells of a 96-well plate and grown as indicated in Methods. After 48 hours treatment with 2 μM FTI-277 (FTI-277) or no treatment (NT), the cells were fixed and stained with an antibody recognising aurora A (αIAK1; BD Biosciences) and the cell cycle distribution was calculated based on the total intensity signal in the Hoechst channel within the nuclear area using the ScanR analysis software. Ten wells were considered per sample and 12 images were acquired randomly in each well. **A**, Representative cells falling within the G2/M gate of treated (FTI-277) or untreated (NT) HeLa cells. **B**, The graph shows the percentage of G2/M phase cells that have Aurora A localized in spots at centrosomes in FTI-277-treated cells and in untreated cells. Two hundred randomly chosen cells were counted for each condition.

To determine if FTI-277 changes Aurora A activity/localization, HeLa cells were seeded in 96-well plates, left to adhere and then grown for a further 48 hours in the presence of 2 μM FTI-277 or in mock medium as described in Methods. The localization of Aurora A was then evaluated in ten wells by determining the Aurora A localized on or near nuclei using FITC-fluorescently labeled Aurora A antibody on fixed cells (Figure 6). The analysis was performed using the ScanR automated microscopy and software platform (Olympus) that allows a multiparametric analysis of fluorescence images and their statistical analysis.

We set masks that allow the identification of Aurora A spots at the centrosomes as well as the determination of the mean intensity FITC (Aurora A) and Hoechst (DNA) channel within the nuclear and cytosolic area. Data analysis showed that FTI-277 treatment of HeLa cells results in a marked reduction of Aurora A localization at centrosomes during the G2-M phase (Figure 6A and 6B), a stage at which Aurora A expression peaks [47], with only 32% of G2-M cells (200 cells counted for every condition) showing Aurora A localized to centrosomes in FTI-277-treated cells compared to 52% in untreated cells. The Aurora A localization in FTI-277-treated cells was generally more diffuse over the whole cell. This difference could not be observed over the entire population of cells, as the cells were not synchronized and were mainly in G1, when Aurora A is difficult to detect [47]. These observations support the array data and imply that FTIs can affect chromosome segregation by impacting on Aurora A degradation and/or localization.

### Cell cycle analysis of FTI-277-treated HeLa and MCF-7 cells

Progression through the cell cycle is a carefully regulated process in all eukaryotes. Periodic activation of cyclin-dependent kinases (CDKs) is required to pass through START and thus for commitment to the next round of cell division. The comparative analysis of the protein networks acting in the cell cycle also suggested that FTIs might impact on entry into the cell cycle, possibly by acting on G1 cyclins and/or on the cyclin-dependent-kinase Cdc28 as these proteins were at the hub of genes deregulated in FTI-treated and in *Δram1 *cells.

Thus, we further studied the effect of FTI-277 on cell cycle entry and progression in HeLa and MCF-7 cancer cell lines using image analysis and the ScanR platform to measure Hoechst total intensity distribution throughout the cell cycle in 5 μM FTI-277-treated and untreated cells. As above, the effects of FTI-277 on cell cycle progression were analysed in 96-well plates, where cells were grown for 48 hours in the presence (FTI) or absence (NT) of FTI-277. After fixation, nuclei were stained with Hoechst and the percentage of cells in the G1, S or G2 phase of the cell cycle was calculated based on the total intensity Hoechst signal present within the nuclear region using the ScanR analysis software. More than 850 cells, seeded in a total of five wells, were counted for each experiment and the mean of two independent experiments were considered.

HeLa and MCF-7 cells have different degrees of sensitivity to FTI-277, MCF-7 being the most sensitive [7,48]. The results show that FTI-277 treatment affects the cell cycle distribution of both HeLa and MCF-7 cells but in an opposite fashion. Approximately a 2% increase in the G1 population, compared to the untreated control, was observed in MCF-7 cells while a comparable decrease was observed in HeLa cells (Additional file 2: Figure S6). The G2 population in both cell types showed the expected reciprocal changes (Additional file 2: Figure S6A). These data indicate that FTI acts at either the G1/S or G2/M transition depending on the cell type.

To gain further insight into the G2/M phenotype of HeLa cells, we measured histone H3 phosphorylation at serine 10 using a phosphospecific antibody (αPhoH3, Additional file 2: Figure S6C). Histone H3 has a key role in the folding and inter-association of the chromatin fibers prior to and during mitosis. Entrance into mitosis is accompanied by hyperphosphorylation of Ser-10 of histone H3. Mitogenic stimuli, mediated by Ras activation, are also accompanied by an increase in Ser-10 phosphorylation of H3. Under the conditions used in this study, only a minor proportion of the total cellular population was found to be in mitosis. However, an increase of 16% in the number of mitotic cells, relative to untreated cells, was observed in HeLa cells. No such effect was detectable in MCF-7 cells treated in a similar manner in independent experiments (Additional file 2: Figure S6B).

**Figure 7:**
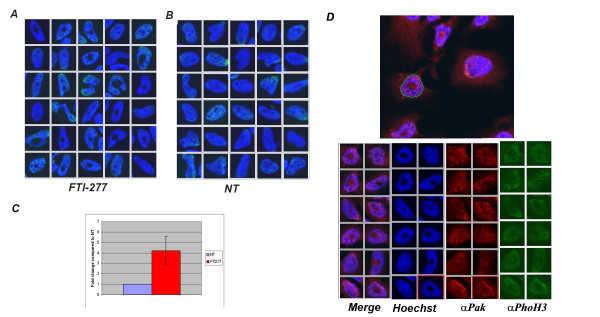
**Nuclear morphology in HeLa G1 cells treated with FTI-277**. The nuclear morphology of HeLa cells, grown and treated as described in Methods, was analysed throughout the cell cycle by plotting the mean intensity signal in the Hoechst channel (x) over the nuclear area (y) using the ScanR analysis software. G1 cells with an altered nuclear morphology were identified in FTI-treated samples and gated to define the mean intensity and area coordinate of this population. A gallery of images of this population is shown in **(A)**. The same region of untreated (NT) cells is shown in **(B)**. **C**, the fold change of G1 cells with an altered nuclear morphology in FTI-277 treated versus NT cells present in the gated population was calculated and plotted in the graph; the mean and the standard deviation of two independent experiments are given. **D**, the upper panel shows the software mask that identifies the nuclei in the images. The lower panel shows galleries of nuclei in FTI-277-treated cells present in the gated population, stained with Hoechst to label nuclei and with anti-Pak (αPaK) and anti-Phospho-histone H3 (αPhoH3) antibody as indicated

A careful inspection of the nuclear morphology of HeLa cells throughout the cell cycle, by plotting the mean intensity values of the Hoechst signal in the nuclear area, identified a subpopulation of cells within the G1 population with an altered nuclear morphology in FTI-treated HeLa cells (Figure 7A). Although cells with a similar area and mean intensity values of the Hoechst signal are also present in the untreated (NT) population (Figure 7B), they are few in number compared to the FTI-treated cells (Figure 7C) and have no nuclear morphological defects (compare respective galleries in Figure 7A and 7B). The FTI-treated cell population with nuclear morphological defects appears to have an altered chromatin distribution within the nuclei, DNA staining being absent over a large area within the nucleus. Proper mitotic chromosome condensation is essential for the correct segregation of sister chromatids into two daughter cells. Typically, chromatin condensation becomes apparent in prophase and is maximal during the stages of mitosis. As mentioned previously, Histone H3 Ser-10 phosphorylation is a signature of mitotic entrance. The presence of a defect in chromatin distribution in treated HeLa G1 cells could be therefore indicative of a premature activation of histone H3 at G1 and be the cause of the accumulation of G2 cells. Therefore, we measured the level of activation of histone H3 within the population with an altered morphology and compared it to the intensity values observed in the total population. No major changes were observed in the mean intensity values of αPhoH3 present in this population compared to the total population (Figure 7D, αPhoH3 panel). Thus the chromatin distribution defects in FTI-treated HeLa G1 cells is unlikely to be due to a defect in chromatin packaging dependent on H3 histone activity.

A second possibility is that the nuclei have morphological alterations that impair the normal distribution of uncondensed DNA within the nuclei. We therefore fluorescently immunostained the cytosol and nuclei with an anti-p21 kinase (PAK) antibody (αPak-C19) in the same samples. As can be best appreciated by the merged images in the gallery of cells randomly and blindly chosen by the program among all cells present in the gated population with a morphologically defective area, αPak-C19 distributes equally in normal nuclei and in nuclei with a DNA hole in the centre (Figure 7D, αPAK panel). Although a more careful three-dimensional analysis of these cells is required, these data suggest that nuclei have morphology defects that cause the chromatin distribution defects observed.

In summary, FTI treatment in yeast as well as in mammalian cancer cell lines affects chromosome segregation by altering Aurora A localization and reduces progression through the cell cycle in HeLa and MCF-7 cells. High content analysis of the images obtained from HeLa cells show that a significant proportion of the G1 cell population (about 6%) experiences chromatin distribution defects that do not involve H3 hyperphosphorylation or distribution changes but are more likely due to nuclear envelope morphology defects.

## Conclusions

A decade of preclinical and clinical studies has shown that chemically different FTIs act in a well-defined mechanistic-driven manner but the signalling modules that are affected by FTase down-regulation have remained unclear so far [5,8]. Here we have shown that FTI peptidomimetics affect the chromosome segregation machinery at the level of the kinetochore and Aurora A mis-localization is one of the features of FTI-treated cells. This is expected to result in replication defects that might account for the overall FTI antiproliferative action.

A second target of FTI-induced transcriptional changes (up- or down-regulation) are key downstream regulators of transcriptional responses that control ribosomal expression and cell cycle progression mediated by TORC1/Sch9/S6K1 signalling pathways. This effect was observed in both FTI-treated and *Δram1 *cells indicating that FTase down-regulation is *per se *a "cellular stress signal" that is monitored by a specific intracellular machinery that impinges on the TORC1/Sch9/S6K1 signalling pathways. Further data are required to conclusively show that this is the case but the data reported here strongly support this view.

The PI3K/Akt/mTOR pathway is a survival pathway often constitutively activated in many types of cancer [35]. Indeed, a synergistic action of rapamycin and clinically relevant FTIs has been previously observed [3]. Thus, an effect of FTI in down-regulating mTOR signaling might account for its antiproliferative action in malignancies in which TORC1/S6K activation plays an important role. That this is the case is also suggested by the finding that the clinically-relevant FTI, SCH66336 (lonafarnib), inhibits mTOR signaling. It has been suggested that this might be due to defective RheB farnesylation upon FTI treatment [39]. The fact, however, that the yeast RheB homologue does not appear to act upstream of TOR1 suggests that the transcriptional deregulation of TOR-downstream effectors as seen in this study does not depend on poor prenylation of a yeast RheB-like protein.

Interestingly, many genes transcriptionally deregulated in FTase-deficient cells belong to the category of tumor suppressors. These observations support the view that FTI treatment might result in different responses depending on the role that PTEN or the DLC-1 tumor suppressor has in a given cancer pathology.

Finally, it is noteworthy that the up-regulation of the multidrug resistance machinery occurs solely in FTI-treated cells. Due to the high number of MDR and ABC transporter genes in mammalian cells, as well as uncertainty as to the direct orthologues of the yeast Pdr5 and Pdr1 genes, it was not possible to directly reciprocate this finding in mammalian cells. However, supporting our array and image analyses, a marked up-regulation of ATP11a but also decreased expression of the ABC transporter ABCA1 has been associated with SCH66336 resistance in murine lymphoblasts [24]. Others have reported that Tipifarnib, another FTI used in clinical trials, has an inhibitory effect on MDR transporter activity via an as yet unknown mechanism [49]. Curiously, however, FTI-277 reduces endogenous expression of MDR1 in the human colorectal cancer cells HCT-15 and SW620-14 [28]. All together, the yeast and mammalian data suggest that FTI inhibitors impact on the multidrug resistance response at different levels and different members of the ABC transporter superfamily are involved. Thus, the MDR response has to be carefully evaluated case-by-case upon FTI treatment.

In conclusion, this study shows that FTase inhibition activates, in yeast and in mammals, a well-defined transcriptional response. We propose that defects in FTase activity are *per se *a cellular stress, normally monitored by Ras/PKA and TOR/Sch9/S6K1-responding genes. Furthermore, this study shows that even suboptimal concentrations of FTI drugs can boost the detoxification pathways leading to MDR up-regulation and thus to FTI resistance.

## Methods

### Yeast strains, drug compounds, plasmid constructs, media and growth conditions for yeast cells

Strains, plasmids and oligonucleotides are listed in Table 1, Table 2 and Table 3, respectively. BY4741 and YDL090C Δ*ram1 *(isogenic with BY4741) strains were purchased from EUROSCARF, the K699, W303 and *Δras2 *strains were a gift from Prof. Kim Nasmyth (University of Oxford). Media, transformation and genetic manipulation as well as molecular procedures used were previously described [50]. Unless otherwise specified, yeast cells were grown at 27°C with agitation in YPD medium or SD medium lacking the appropriate amino acids for plasmid selection as previously described [50]. FTase inhibitor I (Cat.N 344510), FTI-277A (Cat. No. 344555), GGTI-298 (Cat. No. 345883) and Manumycin A (Cat.N 444170) were purchased from Merck-Calbiochem (see http://www.merck-chemicals.it/life-science-research for product specificity). GFP-Ras2pUG34 was constructed by polymerase chain reaction (PCR) using the High Fidelity TAQ polymerase (Roche) and the oligonucleotides Ras2Fw and Ras2Rv listed in Table 3. The PCR fragments were purified, digested with BamHI and EcoRI and cloned in the same sites of vector pUG34 as previously described [50].

**Table 3:** Oligonucleotides list

Oligo name	Oligo sequence 5'-3'
Ras2 Fw	AAAGTGGATCCAAAATGCCTTTGAACAAGTCGAAC

Ras2 Rv	AATAGAATTCTTAACTTATAATACAACAGCCACCC

Ybr159w Fw	TTTGGTGGTCTGATTCCCA

Ybr159w Rv	TCCCCAGCCAAAGAGTTAGA

Yor358w Fw	TTTTCGCCAAAGCCTGTGA

Yor358w Rv	CAGGGCCTCTGCGATATCTG

Ydr180w Fw	CCGCTAGCGCAGAGTGTTCTA

Ydr180w Rv	TCACACTTTCGAGCTTATGCTTTG

Ymr094w Fw	GAAAACTCCTCACCTAGCTCCACA

Ymr094w Rv	TTGTGCGTTGCTCCCACA

Ygl249w Fw	AATGCAGCACACTAACGGAGG

Ygl249w Rv	GATTTCTCAGGTGAGATCCGTTG

Ygl238w Fw	GTATCAAGCCGTTGGCACAAC

Ygl238w Rv	CTTTAGTAGCCTTGTCACGGCAG

Ymr195w Fw	CCAAGCCATGAATAACGTCCA

Ymr195w Rv	GGCATAAAGATCGGTCAATTTATCA

Ylr057w Fw	TCAAACCGCATATTACAACGGAC

Ylr057w Rv	TCTACCTTGTTGACCCAAAATGG

Ygr110w Fw	CCCCAGCAGGCGTGTCT

Ygr110w Rv	TCTGTCCCAAAGCTTGACATACC

Ypl034w Fw	ATGGGTGTGATCCAACTGGAA

Ypl034w Rv	GATGGTAGCTCCAATACAGGCAC

Ydr252w Fw	GATTCATGGCCAACCGAAAG

Ydr252w Rv	GTTATGAGCCAAACCTGTCAAATACTC

### Fractionation of cell extracts

Cells were grown in the presence or absence of 10 μM FTase inhibitor I (Merck-Calbiochem) in SD-HIS as described in the text. The FTI was added to exponentially growing cells at OD_600 _= 0.2 and the cells were harvested at OD_600 _= 0.6. Crude extract preparation using glass beads, fractionation by differential centrifugation and Bradford assay to estimate protein concentration were performed as previously described [50]. Briefly, fractionation of crude extracts was carried out by centrifugation at 15.400 × g for 30 minutes at 4°C. The resulting P15400 × g fractions was resuspended in buffer I (10 mM Tris-HCl pH 8.0, 1 mM PMSF, 1 tablet protease inhibitor, [Roche]) and then in 2× SDS sample buffer (20% glycerol, 2% SDS, 125 mM Tris-HCl pH 6.8, 0.1 mg bromophenol blue) and boiled prior to separation by SDS-PAGE and processed for immunoblotting. Anti-GFP antibody (Roche) was used to detect GFP-Ras2. Western blotting, immunodetection and ECL detection (SuperSignal, PIERCE) and exposure to X-ray films (KODAK-AR) were performed as previously described [50]. Results were analysed and quantified on a densitometer Pharos FX using the Quantity One software (BioRad). When indicated the amounts of proteins transferred on the nitrocellulose membrane were determined with Ponceau S staining as previously described [51].

### Fluorescence microscopy in yeast cells

Typically, cells expressing the indicated GFP-tagged protein were grown to stationary phase overnight in the appropriate selective SCD media as previously described [50]. The cultures were then re-inoculated in fresh media at OD_600 _= 0.1 and grown with shaking at the temperature indicated in the text. The indicated FTI was added (treated) or not added (control) at OD_600 _= 0.2 (T0). Samples were then taken at the indicated time points. At each time point cells were harvested by centrifugation and, unless otherwise indicated, immediately inspected. Images were taken with a Nikon TE 2000 inverted microscope equipped with a 60× objective NA1.4 and CCD camera using the appropriate filters.

### Microarray design, acquisition and analysis

*Array source and experimental design*: Yeast Type 6.4k4 arrays (University Health Network http://www.microarrays.ca) were used. These are double spotted glass arrays with 6,240 different yeast ESTs and 160 Arabidopsis genes as controls. All materials spotted are in the form of double stranded DNA and are coupled to the slide matrix. The DNA is derived from PCR amplification of synthetic EST clones. Type of Array: Yeast; Probe Set Arrayed: Y 6.4k4; Arrayer Used: SDDC24; Slide Batch Number: 701; Slide Batch Code: 24030514; Slide Type: Corning CMT-GA; Manufacturer Lot Number: 29102000. Further information on array design is available at the manufacture's web site (http://www.microarrays.ca/products/microarrays_layouts.html. *Microarray acquisition, normalization and visualization*: Axon ScanArray 4000 and the Genepix version 6.0 (Molecular Devices) was used to acquire images, quantification, gene mapping, normalisation and chip quality control. Data normalization included a manual erase of the spots corresponding to "dirty" spots on the images. Moreover, we excluded the spots with a high percentage of saturated pixels and with a weak signal over background, and all spots flagged absent or bad. All data were deposited at the ArrayExpress Repository at EBI http://www.ebi.ac.uk/microarray-as/ae/ with accession number: E-MTAB-215, E-MTAB-216 and E-MTAB-217 for the expression profile of *S. cerevisiae *after FTI treatment, genetic block of FTase and GGTI298 treatment, respectively. The Gene@work software (IBM research; http://www.research.ibm.com/FunGen/FGGenesAtWorkDoc.html#Visualizing) was used to visualize the expression levels in each array. The color plots derived from each array are given in Additional file 2: Figure S1, S2, S3 and the respective Figure legends in Additional File 1). Briefly, cDNA microarray measurements are made using the two color fluorophores Cy3 and Cy5, where one color corresponds to the control and the other to the sample of interest. Dye swapping method was used to minimize the effects of the differential incorporation of these two dyes into the cDNAs derived from the mRNAs. The measured values are reported as the logarithm base 2 of the ratio (*log*_*2 *_*ratio) *of the two channels. The input data of the color plots in Additional File 2 figures S1, S2 and S3 are .txt files containing the *log*_*2 *_*ratio *of medians of the intensity signals from treated and untreated samples. These were calculated by the Acuity software (Molecular Devices) from the lowess normalized .gpr files obtained from scanning the microarrays by Gene Pix (Molecular Devices). Genes flagged absent (too low intensity over background) or flagged bad were excluded from the calculations.

*Array statistical analysis and pattern recognition*: after acquisition by using GenePix6 software the .gpr data generated were loaded onto the Acuity software (Molecular Devices) for lowess normalization and T-test statistical analysis. Four arrays for FTI, five arrays for *Δram1 *and eight arrays for GGTI-298 were used for statistical analysis. Only the spot signals that were present in at least 60% of the analysed microarrays were considered. The *log*_*2 *_*ratio *between the medians of the treated and control and the mean of the *log*_*2 *_*ratio *among the various arrays was calculated. Only genes having *a log*_*2 *_*ratio *corresponding to at least 0.5 fold over- or under-represented relative to control were considered differentially expressed (Additional files 1 and Additional file 2 tables). These data were finally statistically filtered using Acuity software T-test analysis. All genes with a p-value > 0.2 were excluded to have at least 80% confidence in T-test analysis. The hits were finally clustered according to the features described in the text by using internet tools freely available at SGD http://www.yeastgenome.org/GOContents.shtml and the default search value described therein.

### RNA extraction and cDNA labelling

*Growth conditions for RNA extraction*: Treated and untreated cells were grown in parallel cultures, with equal-size flasks and with the same stock of media, to ensure that experimental growth conditions were equal for each pair of tested strains. Growth conditions, shaking and cell density were carefully controlled over time until harvesting. Typically, cells were grown overnight in a culture tube containing 3 ml YPD. The overnight cultures were then re-inoculated in fresh media to OD_600 _= 0.1 in 100 ml flasks containing 30 ml YPD with (treated) or without (untreated) 10 μM FTase inhibitor I or 10 μM GGTI-298 and harvested at OD_600 _= 0.8 for RNA extraction. *RNA extraction, cDNA labelling and microarray hybridisation: *Total RNA, labelling and cDNA hybridization onto the microarray was performed as described by the array manufacturer http://www.microarrays.ca/info/protocols.html and in Additional file 1. Briefly, RNA extraction was performed following the hot-phenol method [52]. Reverse transcription was performed using Superscript II (Invitrogen) in the presence of either Cy3- or Cy5-dCTP (Amersham) and the manufacture's standard conditions for the other nucleotides. Dye swapping was used to minimise errors due to different incorporation capabilities of Cy3 and Cy5 (Amersham). At least three independent RNA extractions were performed for each pair of tests.

### Quantitative Real-Time PCR

To validate the array normalization and statistical analysis for the FTI arrays we performed quantitative Real-Time PCR. For this, ten genes were randomly chosen among the up- and down-regulated hits of FTase treated cells. As an endogenous control we used the YBR159W gene. Primers were designed using the Primer Express^® ^software from Applied Biosystems. The amplification efficiency of each pair of primers was tested using serial dilutions (5 dilutions) of genomic DNA as template and amplification under standard conditions (see below). Efficiency was calculated as Eff = 10^(-1/slope) ^- 1 × 100. All primer pairs had an efficiency between 90-110%, and showed a single peak in dissociation curves. Primer specificity was also controlled using agarose gel electrophoresis. Total RNA was isolated from yeast cells treated or not with FTase inhibitor I (Merck-Calbiochem). The cells were in the exponential growth phase: the drug was administrated at OD_600 _= 0.2 and the RNA was extracted at OD_600 _= 0.8. Reverse transcription of 2 μg of DNase-treated RNA was performed using random primers and Taqman^® ^Reverse Transcription Reagents (Applied Biosystems) according to the manufacturer's instructions. Amplification reactions were performed in a volume of 50 μl in the presence of SYBR Green PCR Master Mix (Applied Biosystems), 300 nM of each primer, and 2 μl of cDNA on an ABI Prism^® ^7900 HT device. Amplification conditions were 50°C for 2 min, 95°C for 10 min, followed by 40 cycles of 95°C for 15 sec and 60°C for 1 min. Each amplification was run in triplicate, and two independent experiments were performed. Relative expression levels were calculated automatically by the ABI Prism^® ^7900 HT software with the ΔΔCt method using the YBR159W gene as the endogenous control. The up- or down-regulation of five genes has been confirmed by this technique and another four show a similar but smaller response to that determined by microarray analysis.

### Human cell culture, treatments, imaging and cell cycle analysis

The HeLa cell and MCF-7 cell lines were purchased from ATCC. HeLa cells were grown in MEM supplemented with 10% foetal calf serum (FCS), 2 mM L-glutamine, penicillin, streptomycin and non-essential amino acids, at 37°C in 5% CO_2_. Media, serum and reagents for tissue culture were purchased from GIBCO™ (Invitrogen). MCF-7 cells were grown in MEM supplemented with 10% FCS, non-essential amino acids, 1 mg/100 ml insulin, 8.4 mg/100 ml NaHCO_3 _at 37°C in 5% CO_2_.

*HT/HCA Image analysis*: typically 5000 HeLa and 10000 MCF-7 cells were seeded in 96-well Greiner-Bio-One plates and left to attach overnight in their respective media. The indicated amounts of FTI-277 or the vehicle (DMSO) were added and the cells were grown for a further 48 hours. Cells were then washed in 1× phosphate-buffered-saline (PBS) and fixed for 10 min with 4% paraformaldehyde in 1 × PBS, and permeabilized for 30 min in blocking buffer [0.05% saponin, 0.5% bovine serum albumin (BSA), 50 mM NH_4_Cl and 0.02% NaN_3_]. Nuclei were stained with Hoechst (1:1000 in blocking buffer), washed three times and inspected using a 20× objective of the ScanR imaging platform (Olympus). Sixteen images were acquired randomly per well. For cell cycle analysis at least three wells per condition were considered for statistical analysis of the HeLa and MCF-7 cells. For all the others five wells were considered.

Determination of mitotic cell number: fixed cells were incubated for 75 min with α-PhosphoH3 pSer10 (Sigma) diluted 1:100 in blocking buffer, washed three times in 1 × PBS, and further incubated for 30 min with anti-mouse Alexa488 conjugates (Molecular Probes) diluted 1:500 in blocking buffer, washed three times and inspected using the ScanR microscope (Olympus). For determination of Pak1/2/3 localization, an anti-Pak C19 antibody (Santa-Cruz) and anti-rabbit Alexa546 were used.

For determination of Aurora A localization, an anti-IAK1 (BD Biosciences) and anti-mouse Alexa488 conjugates (Molecular Probes) were used. HeLa cells were treated for 48 hours with 2 μM of FTI-277 or with the vehicle, and then fixed using the procedure indicated above.

To determine the phosphorylation of ribosomal protein S6, an Alexa555-conjugated anti-PhosphoS6 antibody (Cell Signaling) was used. Typically 4000 HeLa and 8000 MCF-7 cells were seeded in five wells per experimental condition. Cells were left to attach overnight, and treated with 5 μM of FTI-277 or with the vehicle for 24 hours in starvation medium (0.1% FCS). Cells were fixed, washed and probed with Hoechst and processed for ScanR acquisition as described above. Quantification and statistical analysis were performed with the in-built ScanR analysis software (Olympus).

## Competing interests

The authors declare that they have no competing interests.

## Authors' contributions

GP and ARW performed the experiments and data analysis. DDG performed the cell culture and provided technical assistance. CW performed the RT-PCR and critically reviewed the manuscript. GP and ARW wrote the paper. All authors read and approved the manuscript.

## Supplementary Material

Additional file 1 **Additional methods and additional data figure legends: detailed information of the methodology used for microarray acquisition and analysis; figures and tables legends of data shown in Additional file **2. Click here for file

Additional file 2 **Additional figures and tables: supplementary tables, figures and graphics of the expression profiling results**. Click here for file
