# Supplementary material for: A yeast-based genomic strategy highlights the cell protein networks altered by FTase inhibitor peptidomimetics
Source: Mol Cancer. 2010 Jul 23;9:197. doi: 10.1186/1476-4598-9-197 (PMC2925370; doi:10.1186/1476-4598-9-197)
Supplement: Additional file 1 — Additional methods and additional data figure legends: detailed information of the methodology used for microarray acquisition and analysis; figures and tables legends of data shown in Additional file 2. [file 1476-4598-9-197-S1.DOC]

**Additional File 1**

**Methods**

**Details of Methods of RNA extraction, labeling and hybridization used for microarray analysis**

Total RNA, labelling and cDNA hybridization on the microarray were performed as described by the array manufacturer (University Health Network/Ontario Cancer Institute (OCI) Microarray Centre). Detailed protocols are available at: http// [www.microarrays.ca](http://www.microarrays.ca/).

RNA extraction was performed following the hot-phenol method [52]. After acid phenol extraction, total RNA was precipitated overnight using 3 M Na acetate pH 5.3 and ice-cold 100% ethanol. The total RNA pellet was then washed with ice-cold 70% ethanol and finally pelleted and re-dissolved in distilled water.

cDNA preparation and Labelling: Cyanine Dye swapping was used to minimise errors due to differential incorporation of Cy3 and Cy5. Total RNA prepared as described above was combined on ice (and in the dark) with 8 μl of 5X First Strand reaction buffer (supplied with reverse transcriptase Superscript II, Invitrogen) 1.5 μl mRNA primer (5’-T_20_ VN, 100 pmol/μl), 3 μl 20mM ddNTP (6.67 mM each of dATP, dGTP, dTTP), 1 μl 2mM dCTP, 1 μl 1 mM Cyanine 3 or Cyanine 5 dCTP (Amersham), 4 μl 0.1M DTT, 20 μg total RNA (treated or control), H_2_O to 40 μl. The samples were incubated at 65°C for 5 min, then for 5 min at 42°C. 2 μl of Reverse Transcriptase Superscript II, (Invitrogen) were added. The samples were incubated at 42°C for 3 hours. Then 4 μl of 50 mM EDTA (pH 8.0) and 2 μl of 10 N NaOH were added. After 20 min at 65°C (to hydrolyze the RNA), 4 μl of 5 M acetic acid were added, the 2 samples (control and treated) were united in a single centrifuge tube and the cDNA was precipitated by adding 100 μl of isopropanol and incubating at -20°C for 30 min. The cDNA was pelleted by centrifugation at 15000g, 4°C for 30 min. The pellet was washed with 200 μl of 70% ice-cold ethanol. After centrifugation for 15 min the cDNA was dried in a Speed Vacuum for 5 min and dissolved in 5 μl of H_2_O. Hybridization: 100 μl of DIG Easy Hyb (Roche) and 5 μl of denaturated salmon sperm DNA were warmed at 65°C for 2 min and then cooled to room temperature. 60 μl of the mixture were added to the labelled cDNA and incubated at 65°C for 2 min. The mixture was cooled at room temperature and pipetted onto the array. After placing a coverslip, the array was inserted in a chamber containing DIG Easy Hyb and incubated overnight at 37°C. Washing: after approximately 15 hours the array was placed in 1X SSC to remove the cover slip and incubated with prewarmed 1X SSC + 0.1% SDS at 50°C (moderate shaking) 3 times for 10 min. Then the array was washed six times with 1X SSC (by inversion) and dried by centrifugation.

**Figure and table legends**

**Figure S1. Color plot visualization of the relative gene expression levels of FTI treated versus untreated samples and analysis of the reproducibility of the biological replicates.**

The gene expression data of each microarray is shown as a color plot panel using Gene@Work software (IBM Research, [http://www.research.ibm.com/FunGen/FGGenesAtWorkDoc.html#Visualizing](http://www.research.ibm.com/FunGen/FGGenesAtWorkDoc.html#Visualizing). In the color plots the red-colored spots indicate induction, the green color represent repression, while the black color indicate no change in expression between treated and control samples.

Briefly, cDNA microarray measurements are made using the two color fluorophores Cy3 and Cy5, where one color corresponds to the control and the other to the sample of interest. Usually, the measured values are reported as the logarithm base 2 of the ratio (log_2_ ratio) of the two channels. The input data of the color plots in the figure are .txt files containing the log_2_ ratio of medians of the intensity signals from treated and untreated samples calculated by the Acuity software (Molecular Devices) from the Lowess normalized .gpr files obtained from scanning the microarrays by Gene Pix (Molecular Devices). Lowess normalization was performed by using the Acuity software normalization tools (Molecular Devices). Genes flagged absent (too low intensity over background) or flagged bad were excluded from the calculations.

*The mean of the expression level* for each gene from all microarrays considered in each data set was calculated and shown in the *mean color plot panel.* Analyzing the *FTI array mean color plot,* an overall good reproducibility among different biological replicates for each sample set can be observed: the induced genes (red-colored) are mostly clustered in the upper part of the panel, while the repressed genes (green colored) are clustered in the bottom.

**Figure S2. Color plot visualization of the relative gene expression levels of *Δram1*** **samples and analysis of the reproducibility of the biological replicates.**

See figure legend S1 for the methodology regarding data visualization in color plots. The *Δram1 arrays mean color plot was calculated* and is shown in the right panel. Also in this case an overall good reproducibility among different biological replicates was observed.

**Figure S3. Color plot visualization of the relative gene expression levels of GGTI-298 treated versus untreated samples and analysis of the reproducibility of the biological replicates.**

See figure legend S1 for the methodology regarding data visualization in color plots. The GGTI-298 *arrays mean color plot was calculated* and is shown in the right panel. As in S1 and S2 an overall good reproducibility among different biological replicates was observed.

**Figure S4. Real time validation of microarray data** This graph illustrates the comparison between microarray and real-time PCR expression analysis for 10 randomly chosen genes, using the log_2_ ratio of medians of treated and control samples. The microarray log_2_ ratio of medians is plotted on the x axis, while the real-time PCR log_2_ ratio of medians is plotted on the y-axis. Linear regression analysis was performed and the correlation coefficient (R^2^) is indicated.

**Figure S5. String analysis of FTI down-regulated genes involved in the cell cycle** The graph shows the String analysis - with highest confidence and excluding text mining - of down-regulated genes that are involved in the cell cycle after FTI treatment of BY4741 cells. The arrows indicate the three input genes that are down-regulated, while the hub CLN2 is highlighted.

**Figure S6. FTI treatment effects on cell cycle progression in HeLa and MCF-7 cells**

Cell cycle analysis of FTI277-treated HeLa and MCF-7 cells. **A,** HeLa and MCF-7 cells were seeded in 5 wells of a 96-well plate and grown as described in Methods. After 48h treatment with 5 μM FTI-277 (FTI) or no treatment (NT), the cells were fixed and stained with Hoechst. The cell cycle distribution was calculated based on the total intensity signal in the Hoechst channel within the nuclear area using the ScanR analysis software. Five wells were considered per sample and 16 images were acquired randomly in each well. The *mean of the total intensity* signal calculated for five wells on all images was plotted in the graph using Excel. Two independent experiments were considered. **B,** mitotic cell determination was performed based on the signal of the FITC-conjugated secondary antibody recognising the anti-PhosphoH3 pSer10 antibody (PhoH3) on fixed cells treated or not treated as indicated above. The fold change of αPhoH3 in FTI-277 treated cells versus not treated cells (NT) was calculated and plotted in the graph. **C,** images of the αPhoH3 positive signal and the software mask used are shown (upper and lower panels). Hoechst staining of the cell population and the PhoH3/Hoechst merged signal are shown in the middle panels.

**Table S1.** RT-PCR validation of microarray data. Ten genes were randomly selected for RT-PCR. The fold change (expressed as log_2_ ratio of medians of treated and control samples) of the RNA transcripts in microarray data and RT-PCR is indicated in the table together with the gene identifier and the gene name.

**Table S2.** List of statistically significant (p-value <0.2) gene transcripts up- and down-regulated at least 0.5 fold in 10 μM FTase Inhibitor I-treated versus untreated cells. The gene name, the log_2_ ratio of medians of treated versus untreated and the respective p-value are shown for each gene.

**Table S3.** Classification of the 69 up-regulated genes of FTI-treated cells binned by compartment localization using Super GO-Slim Component at SGD. The GO Slim Mapper bins group of genes into broad categories, i.e. GO Slim terms.

**Table S4.** Classification for biological process of the 17 up-regulated genes that reside in the nucleus in FTI-treated cells. They were identified by binning them using GO Term Binning by Process at SGD.

**Table S5.** Compartment ontology of the intra-nuclear localized up-regulated genes - upon FTI treatment - that reside in the nucleus, determined by binning these genes using GO Term Binning by Component at SGD.

**Table S6.** Classification by biological process of the 69 significantly up-regulated genes upon FTase inhibitor I treatment, determined by binning these genes by using Super GO-Slim Process clustering at SGD.

**Table S7.** Classification by biological process of the 35 significantly down-regulated genes upon FTase inhibitor I treatment, determined by binning these genes by using Super GO-Slim Process clustering at SGD.

**Table S8.** List of statistically significant (p-value <0.2) gene transcripts up- and down-regulated at least 0.5 fold upon RAM1 deletion. The gene name, the log_2_ ratio of medians of treated and control and the respective p-value are shown for each gene.

**Table S9.** Classification of the 99 up-regulated genes upon RAM1 deletion binned by compartment localization using Super GO-Slim Component clustering at SGD.

**Table S10.** Classification by biological process of the 99 significantly up-regulated genes upon RAM1 deletion, determined by binning these genes using Super GO-Slim Process clustering at SGD.

**Table S11.** Classification by biological process of the 38 significantly down-regulated genes upon RAM1 deletion, determined by binning these genes using Super GO-Slim Process clustering at SGD.

**Table S12.** List of statistically significant (p-value <0.2) genes up- and down- regulated at least 0.5 fold upon 10 μM GGTI-298 treatment. For each gene are indicated: the gene name, the log_2_ ratio of medians of treated versus untreated and the respective p-value.

**Table S13.** Classification by compartment localization of the 86 significantly up-regulated genes upon GGTI-298 treatment, determined by binning the up-regulated genes using Super GO-Slim Component clustering at SGD.

**Table S14.** Classification by biological process of the 86 significantly up-regulated genes upon GGTI-298 treatment, determined by binning these genes by using Super GO-Slim Process clustering at SGD.

**Table S15.** Classification by biological process of the 20 up-regulated genes - upon GGTI-298 treatment - that reside in the nucleus, determined by binning these genes using GO Term Binning by Process at SGD.

**Table S16.** Classification by compartment localization of the 95 significantly down-regulated genes upon GGTI-298 treatment, determined by binning the down-regulated genes using Super GO-Slim Component clustering at SGD.

**Table S17.** Classification by biological process of the 19 down-regulated genes - upon GGTI-298 treatment - that reside in the nucleus, determined by binning these genes using GO Term binning by Process at SGD.

**Table S18.** Classification by biological process of the 10 down-regulated genes - upon GGTI-298 treatment - that reside in the endoplasmic reticulum, determined by binning these genes using GO Term binning by Process at SGD.

**Table S19.** Classification by biological process of the 95 significantly down-regulated genes upon GGTI-298 treatment, determined by binning these genes using Super GO-Slim Process clustering at SGD.
