# Supplementary material for: A yeast-based genomic strategy highlights the cell protein networks altered by FTase inhibitor peptidomimetics
Source: Mol Cancer. 2010 Jul 23;9:197. doi: 10.1186/1476-4598-9-197 (PMC2925370; doi:10.1186/1476-4598-9-197)
Supplement: Additional file 2 — Additional figures and tables: supplementary tables, figures and graphics of the expression profiling results. [file 1476-4598-9-197-S2.DOC]

**Additional File 2**

**Figure S1. Color plot visualization of the relative gene expression levels of FTI treated versus untreated samples and analysis of the reproducibility of the biological replicates**

**
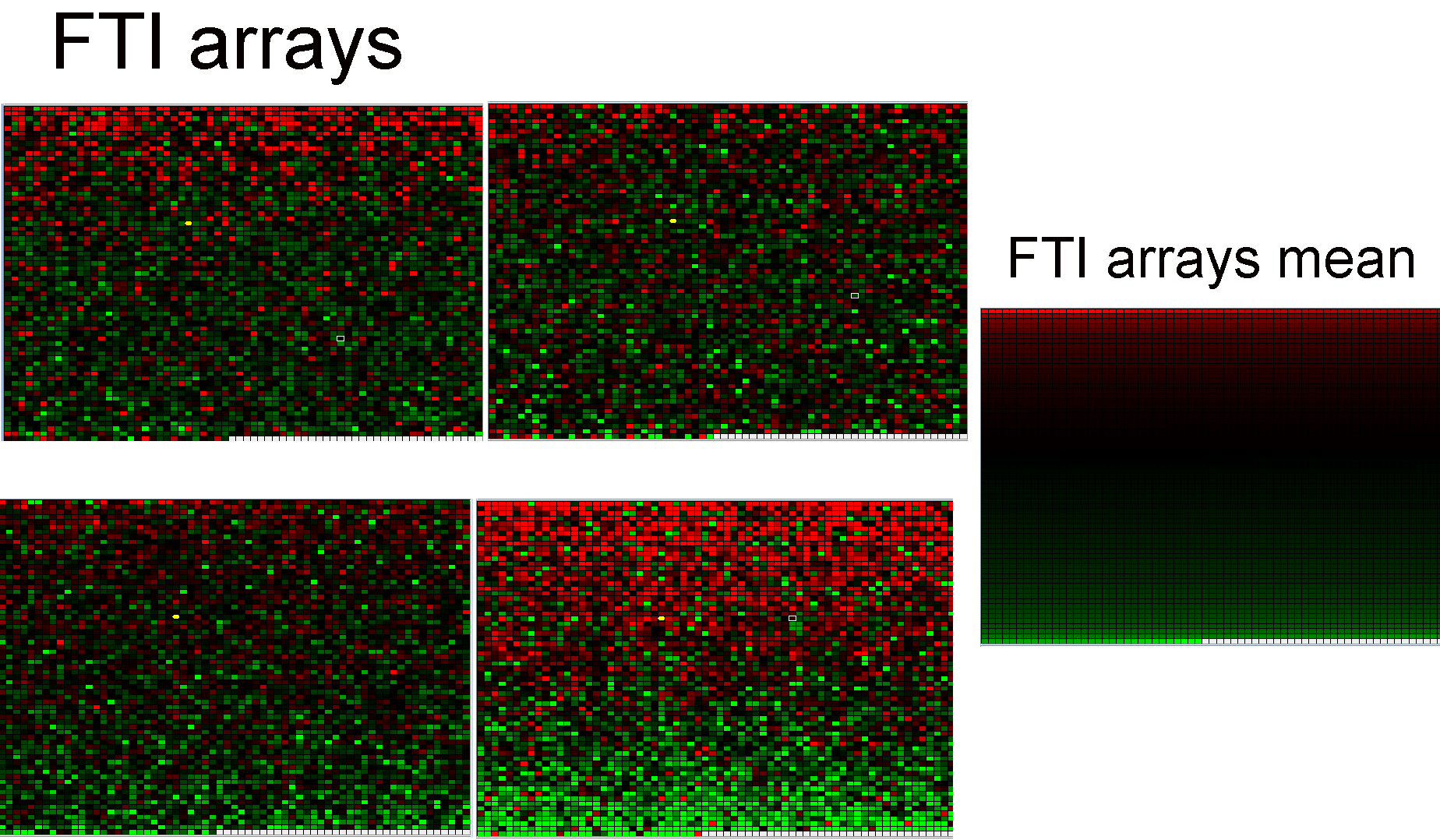
**

**Figure S2. Color plot visualization of the relative gene expression levels of *Δram1*** **samples and analysis of the reproducibility of the biological replicates**

**
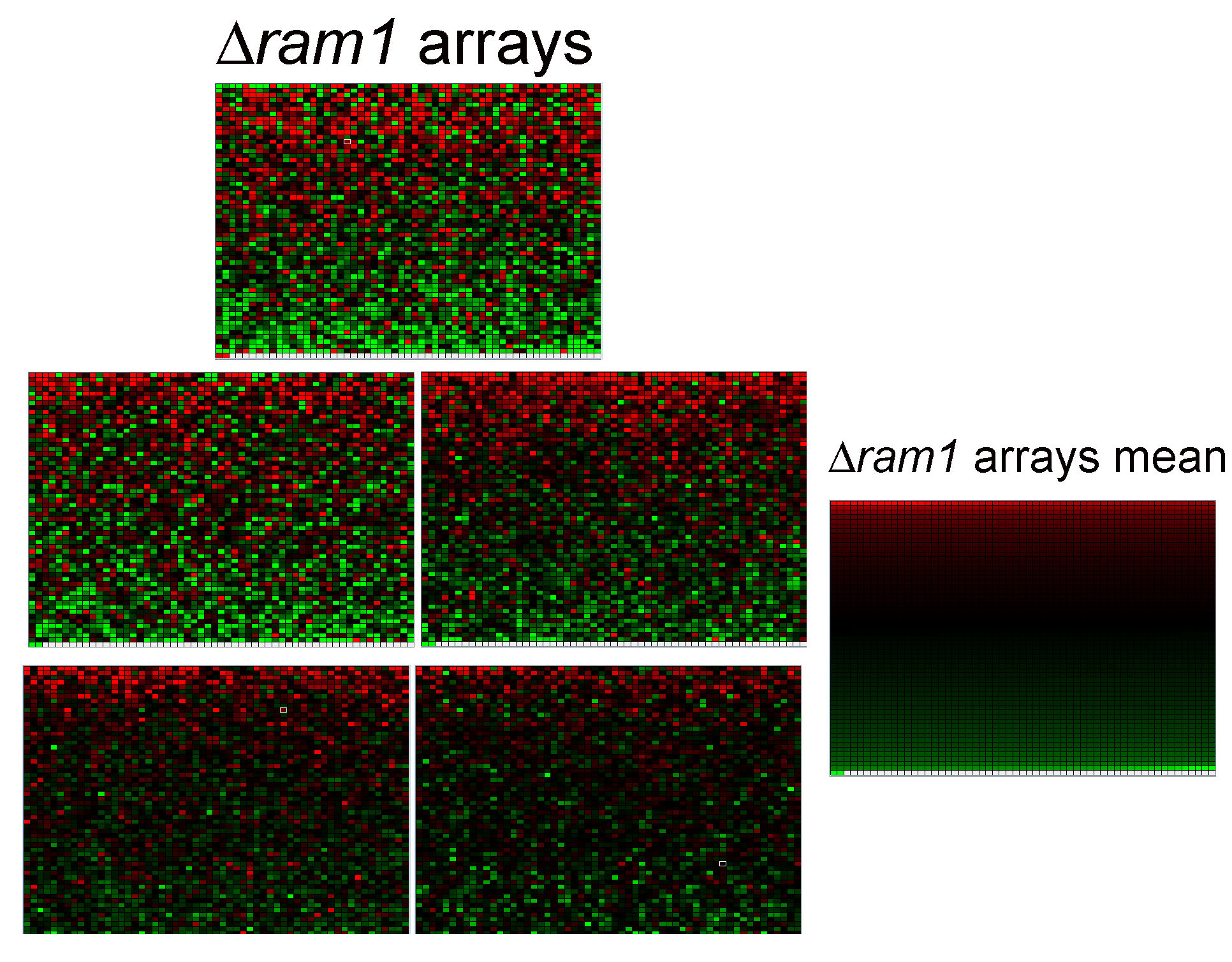
**

**Figure S3. Color plot visualization of the relative gene expression levels of GGTI-298 treated versus untreated samples and analysis of the reproducibility of the biological replicates**

**
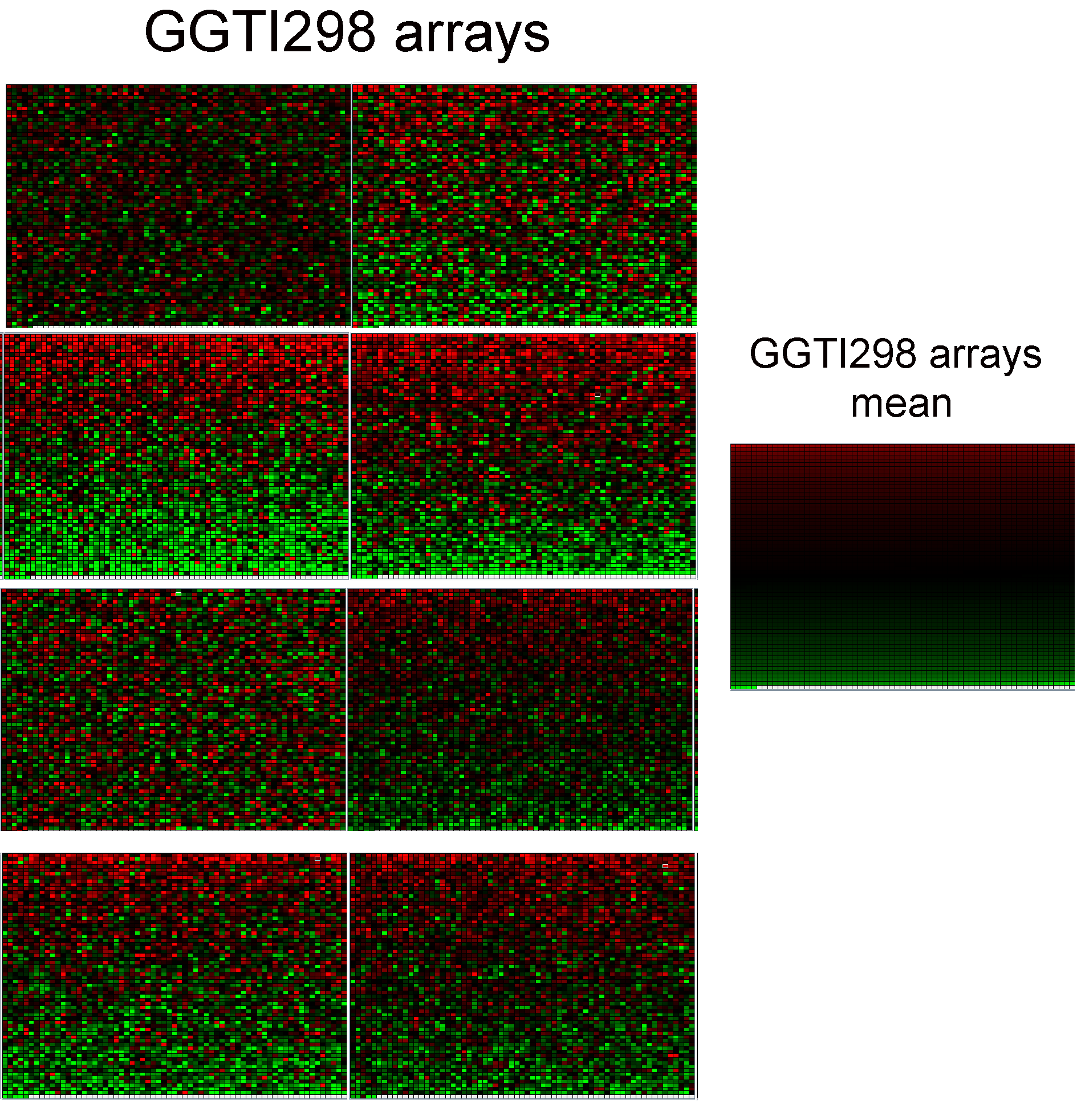
**

**Figure S4. Real time validation of microarray data**


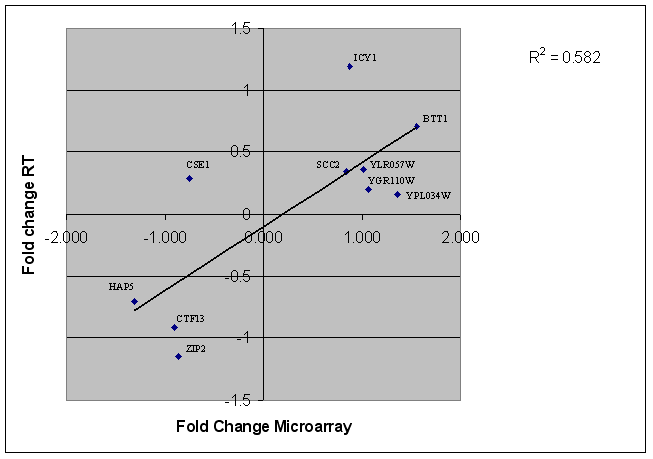


**Figure S5. String analysis of FTI down-regulated genes involved in the cell cycle**

**
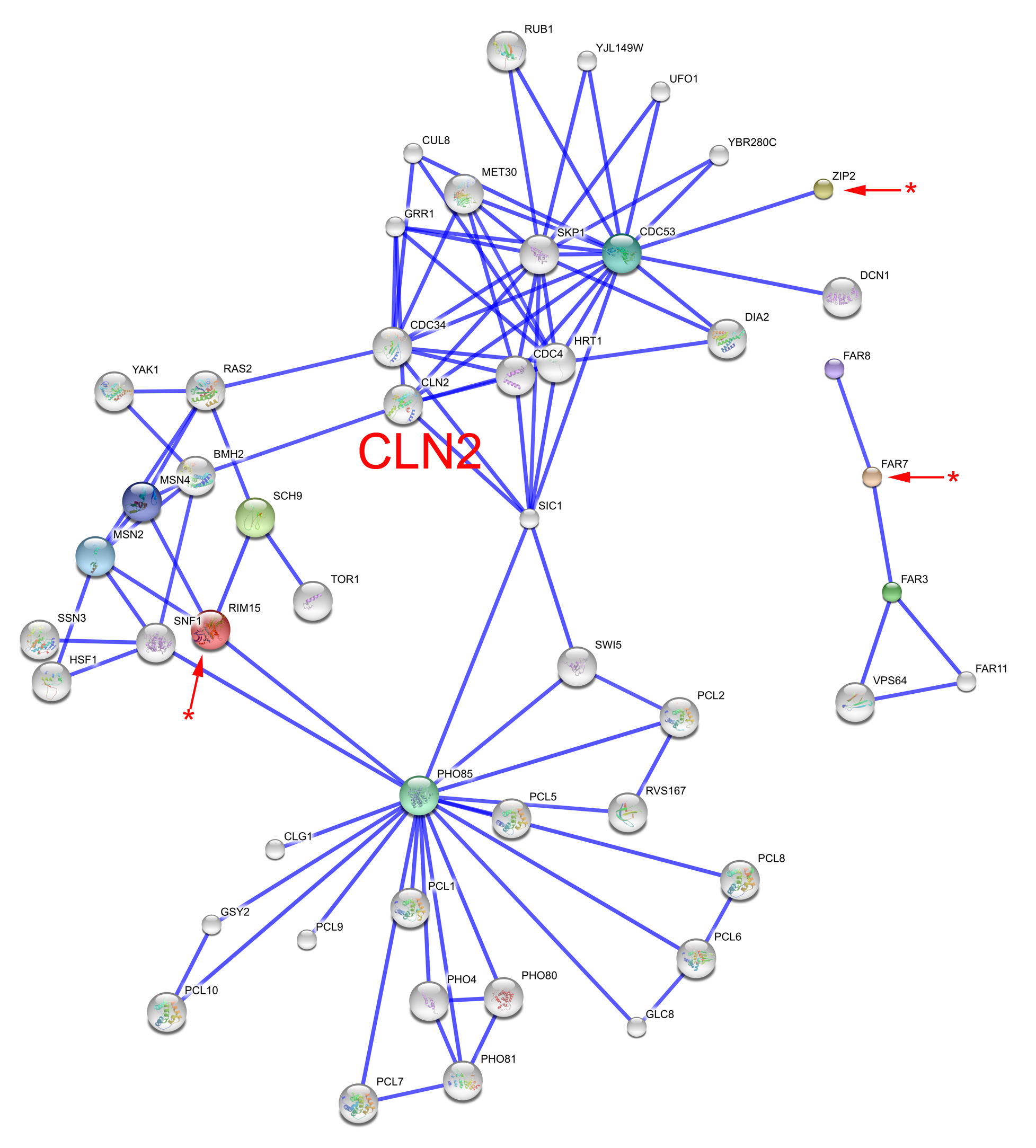
**

**Figure S6. FTI treatment effects on cell cycle progression in HeLa and MCF-7 cells**

**
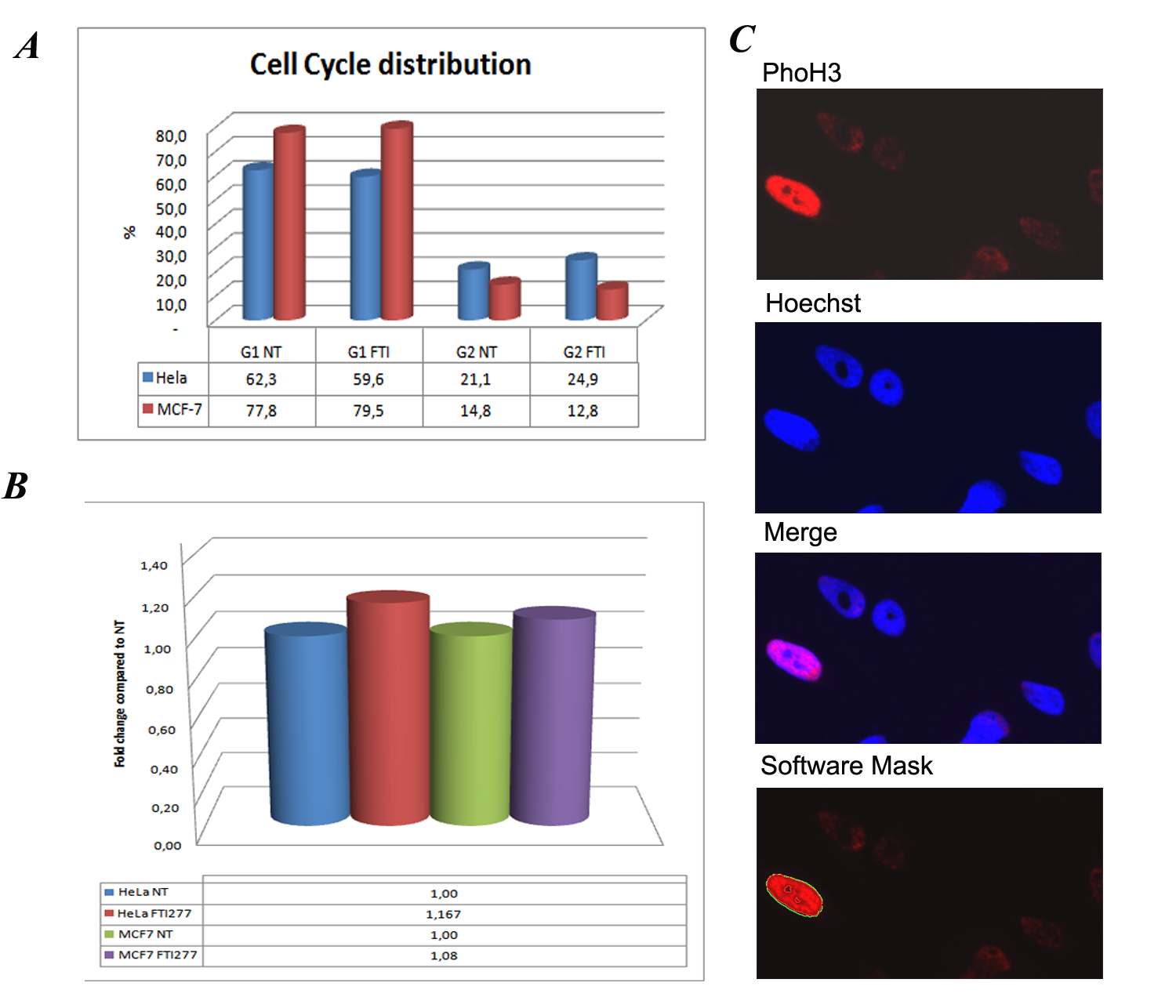
**

|  | |  |  |
| --- | --- | --- | --- |
| **ID** | **Name** | **log_2_ ratio Microarray** | **log_2_ ratio RealTime** |
| **YOR358W** | HAP5 | -1.308 | -0.70 |
| **YMR094W** | CTF13 | -0.906 | -0.92 |
| **YGL249W** | ZIP2 | -0.862 | -1.15 |
| **YGL238W** | CSE1 | -0.754 | 0.29 |
| **YDR180W** | SCC2 | 0.832 | 0.34 |
| **YMR195W** | ICY1 | 0.871 | 1.19 |
| **YLR057W** | YLR057W | 1.006 | 0.36 |
| **YGR110W** | YGR110W | 1.057 | 0.20 |
| **YPL034W** | YPL034W | 1.362 | 0.16 |
| **YDR252W** | BTT1 | 1.558 | 0.71 |

**Table S1. List of genes analysed with RealTime PCR**

**Table S2. List of up-regulated genes in FTI treated cells**

|  | | |  |  | |  |  |  |
| --- | --- | --- | --- | --- | --- | --- | --- | --- |
| **Name** | **log_2_ ratio Treated/**  **Control** | | **p-value** |  | | **Name** | **log_2_ ratio Treated/**  **Control** | **p-value** |
| **UpRegulated** | | |  |  | |  |  |  |
|  |  | |  |  | |  |  |  |
| YAL035C-A | 2.706 | | 0.123017 |  | | YBR285W | 0.717 | 0.196785 |
| YDL038C | 2.651 | | 0.165854 |  | | YDR306C | 0.714 | 0.141818 |
| YLR162W | 1.693 | | 0.073246 |  | | YKL111C | 0.714 | 0.137679 |
| YDR252W | 1.558 | | 0.150644 |  | | YLR137W | 0.712 | 0.037509 |
| YNL337W | 1.502 | | 0.119862 |  | | YKL197C | 0.692 | 0.147029 |
| YBR002C | 1.501 | | 0.186945 |  | | YPL275W | 0.674 | 0.19604 |
| YPL034W | 1.362 | | 0.035125 |  | | YKR075C | 0.674 | 0.099526 |
| YBR085W | 1.231 | | 0.14772 |  | | YJL110C | 0.659 | 0.126074 |
| YDR130C | 1.209 | | 0.144159 |  | | YOR181W | 0.652 | 0.105108 |
| YFR046C | 1.136 | | 0.086442 |  | | YDL210W | 0.649 | 0.13133 |
| YDR286C | 1.096 | | 0.084928 |  | | YNR072W | 0.646 | 0.129528 |
| YNR006W | 1.075 | | 0.130184 |  | | YJR158W | 0.642 | 0.187889 |
| YOR313C | 1.069 | | 0.054136 |  | | YKL159C | 0.629 | 0.168258 |
| YGR110W | 1.057 | | 0.09946 |  | | YDL072C | 0.623 | 0.150906 |
| YDR170C | 1.053 | | 0.062916 |  | | YJR112W | 0.608 | 0.171333 |
| YKL167C | 1.025 | | 0.016986 |  | | YKR073C | 0.603 | 0.103862 |
| YCR014C | 1.007 | | 0.183949 |  | | YNR042W | 0.602 | 0.039612 |
| YLR057W | 1.006 | | 0.175201 |  | | YLR043C | 0.6 | 0.084378 |
| YML054C | 0.998 | | 0.167198 |  | | YDR016C | 0.6 | 0.105436 |
| YBR015C | 0.992 | | 0.146359 |  | | YDL094C | 0.592 | 0.102922 |
| YNL198C | 0.946 | | 0.032954 |  | | YGL109W | 0.59 | 0.009953 |
| YLR145W | 0.933 | | 0.167705 |  | | YLR398C | 0.589 | 0.172335 |
| YDR260C | 0.932 | | 0.012373 |  | | YML083C | 0.589 | 0.013196 |
| YDL246C | 0.925 | | 0.096683 |  | | YDR173C | 0.586 | 0.18437 |
| YKL151C | 0.905 | | 0.10175 |  | |  |  |  |
| YMR195W | 0.871 | | 0.099381 |  | |  |  |  |
| YDR180W | 0.832 | | 0.142602 |  | |  |  |  |
| YDR138W | 0.828 | | 0.190206 |  | |  |  |  |
| YGL069C | 0.827 | | 0.000997 |  | |  |  |  |
| YGL013C | 0.816 | | 0.063056 |  | |  |  |  |
| YMR322C | 0.814 | | 0.03889 |  | |  |  |  |
| YDL009C | 0.806 | | 0.161256 |  | |  |  |  |
| YOL150C | 0.784 | | 0.007677 |  | |  |  |  |
| YBR275C | 0.781 | | 0.026318 |  | |  |  |  |
| YJL102W | 0.774 | | 0.016542 |  | |  |  |  |
| YJR114W | 0.773 | | 0.014246 |  | |  |  |  |
| YBR203W | 0.765 | | 0.167577 |  | |  |  |  |
| YPL134C | 0.758 | | 0.08947 |  | |  |  |  |
| YGL061C | 0.743 | | 0.168394 |  | |  |  |  |
| YHR120W | 0.738 | | 0.116175 |  | |  |  |  |
| YBR057C | 0.735 | | 0.189991 |  | |  |  |  |
| YFR025C | 0.73 | | 0.084491 |  | |  |  |  |
| YJL210W | 0.723 | | 0.016651 |  | |  |  |  |
| YBR211C | 0.721 | | 0.051634 |  | |  |  |  |
| YBL098W | 0.721 | | 0.198862 |  | |  | **List of down-regulated genes in FTI treated cells** |  |
| **Name** | | **log_2_ ratio**  **Treated/**  **Control** | **p-value** | |  | | | |
|  | |  |  | |  | | | |
| **DownRegulated** | | |  | |  | | | |
|  | |  |  | |  | | | |
| YBR113W | | -2.052 | 0.109644 | |  | | | |
| YNL093W | | -1.98 | 0.123258 | |  | | | |
| YJR036C | | -1.532 | 0.04626 | |  | | | |
| YOR358W | | -1.308 | 0.000233 | |  | | | |
| YOR290C | | -1.146 | 0.189811 | |  | | | |
| YKR078W | | -1.045 | 0.131869 | |  | | | |
| YOR242C | | -0.99 | 0.193564 | |  | | | |
| YCR102C | | -0.978 | 0.183539 | |  | | | |
| YLR067C | | -0.925 | 0.091402 | |  | | | |
| YMR094W | | -0.906 | 0.151627 | |  | | | |
| YDR504C | | -0.894 | 0.151864 | |  | | | |
| YGL249W | | -0.862 | 0.154684 | |  | | | |
| YOR190W | | -0.79 | 0.128908 | |  | | | |
| YML010W-B | | -0.79 | 0.057244 | |  | | | |
| YPR080W | | -0.765 | 0.000531 | |  | | | |
| YGL238W | | -0.754 | 0.1857 | |  | | | |
| YPR126C | | -0.751 | 0.125342 | |  | | | |
| YHR079C | | -0.728 | 0.199588 | |  | | | |
| YJL113W | | -0.723 | 0.128948 | |  | | | |
| YIL097W | | -0.702 | 0.084677 | |  | | | |
| YLR374C | | -0.687 | 0.044681 | |  | | | |
| YJR141W | | -0.683 | 0.193068 | |  | | | |
| YLR009W | | -0.663 | 0.065318 | |  | | | |
| YER153C | | -0.658 | 0.182755 | |  | | | |
| YGR219W | | -0.642 | 0.196819 | |  | | | |
| YFL033C | | -0.641 | 0.029482 | |  | | | |
| YPR019W | | -0.618 | 0.130667 | |  | | | |
| YFR008W | | -0.617 | 0.195838 | |  | | | |
| YPL264C | | -0.613 | 0.116504 | |  | | | |
| YIL128W | | -0.611 | 0.034448 | |  | | | |
| YHR073W | | -0.61 | 0.172784 | |  | | | |
| YPR010C | | -0.609 | 0.174474 | |  | | | |
| YNL328C | | -0.607 | 0.020194 | |  | | | |
| YOL095C | | -0.6 | 0.144604 | |  | | | |
| YLR358C | | -0.59 | 0.092722 | |  | | | |

**Table S3. Classification of FTI up-regulated genes according to compartment**

|  |  |  |
| --- | --- | --- |
| **Compartment** | **Frequency (%)** | **Genes Names** |
| cytoplasm | 28 genes, or 40.6% | AAC3, AME1, BNA4, BTT1, COS111, CYB2, DUO1, FIN1, ICY1, LAS17, MEF2, MNN2, MRP49, MSH1, MUM2, ODC1, PEX1, PEX2, RER2, RMP1, SEC7, SKI2, TRX1, UGA4, VPS27, YET3, YKL151C, YKR075C |
| cellular component unknown | 25 genes, or 36.2% | FDH2, SNO4, SOR2, SPS4, YAL034C-B, YBR285W, YDL009C, YDL038C, YDL094C, YDR286C, YGL069C, YGL109W, YGR110W, YJR114W, YKL111C, YKR073C, YLR057W, YLR137W, YLR162W, YML083C, YNL198C, YNL337W, YNR042W, YOL150C, YPL034W |
| nucleus | 17 genes, or 24.6% | AME1, ARG82, CNN1, DAD1, DUO1, FIN1, GZF3, HPR1, NNF1, PDR1, POL4, RIF1, RMP1, SCC2, SKI2, SWM1, YKR075C |
| mitochondrion | 8 genes, or 11.6% | AAC3, BNA4, COS111, CYB2, MEF2, MRP49, MSH1, ODC1 |
| cytosol | 3 genes, or 4.3% | BTT1, SEC7, TRX1 |
| endoplasmic reticulum | 2 genes, or 2.9% | RER2, YET3 |
| can not be mapped to a GO slim term | 5 genes, or 7.2% | HIS2, HXT16, HXT17, RCN1, YDR306C |

**Table S4. Classification of nuclear-located genes in FTI treated cells binned according to biological process**

| **Biological Process** | **Frequency (%)** | **Frequency, on the whole genome, of genes belonging to each Biological Process** | **P-value** | **Genes Names** |
| --- | --- | --- | --- | --- |
| regulation of microtubule polymerization or depolymerization | 3 genes, or 17.6% | 15 genes, or 0.2% | 0.00064 | DAD1, FIN1, DUO1 |
| microtubule polymerization or depolymerization | 3 genes, or 17.6% | 18 genes, or 0.3% | 0.00113 | DAD1, FIN1, DUO1 |
| chromosome segregation | 5 genes, or 29.4% | 139 genes, or 1.9% | 0.00168 | AME1, FIN1, SCC2, CNN1, NNF1 |
| regulation of microtubule-based process | 3 genes, or 17.6% | 25 genes, or 0.3% | 0.00316 | DAD1, FIN1, DUO1 |
| regulation of microtubule cytoskeleton organization | 3 genes, or 17.6% | 25 genes, or 0.3% | 0.00316 | DAD1, FIN1, DUO1 |
| regulation of cytoskeleton organization | 3 genes, or 17.6% | 32 genes, or 0.4% | 0.00675 | DAD1, FIN1, DUO1 |
| regulation of organelle organization | 4 genes, or 23.5% | 93 genes, or 1.3% | 0.00712 | DAD1, FIN1, SWM1, DUO1 |

**Table S5. Classification of nuclear-located FTI up-regulated genes according to compartment**

| **Compartment** | **Frequency (%)** | **Frequency, on the whole genome, of genes belonging to each Compartment** | **P-value** | **Genes Names** |
| --- | --- | --- | --- | --- |
| kinetochore | 5 genes, or 29.4% | 54 genes, or 1.1% | 0.00840 | AME1, CNN1, DAD1, DUO1, NNF1 |

**Table S6. Classification of FTI up-regulated genes according to biological processes**

| **Biological Process** | **Frequency (%)** | **Genes Names** |
| --- | --- | --- |
| cellular process | 39 genes, or 56.5% | AME1, ARG82, BNA4, BTT1, CNN1, COS111, DAD1, DUO1, FDH2, FIN1, GZF3, HIS2, HPR1, LAS17, MEF2, MNN2, MRP49, MSH1, MUM2, NNF1, ODC1, PDR1, PEX1, PEX2, POL4, RCN1, RER2, RIF1, RMP1, SCC2, SEC7, SKI2, SNO4, SOR2, SPS4, SWM1, TRX1, VPS27, YDR306C |
| metabolic process | 29 genes, or 42.0% | AAC3, ARG82, BNA4, BTT1, CYB2, FDH2, FIN1, GZF3, HIS2, HPR1, LAS17, MEF2, MNN2, MRP49, MSH1, MUM2, PDR1, POL4, RER2, RIF1, RMP1, SCC2, SKI2, SNO4, SOR2, SPS4, SWM1, TRX1, YDR306C |
| biological process unknown | 24 genes, or 34.8% | ICY1, YAL034C-B, YBR285W, YDL009C, YDL094C, YDR286C, YET3, YGL069C, YGL109W, YGR110W, YJR114W, YKL111C, YKL151C, YKR073C, YKR075C, YLR057W, YLR137W, YLR162W, YML083C, YNL198C, YNL337W, YNR042W, YOL150C, YPL034W |
| transport | 10 genes, or 14.5% | HPR1, HXT16, HXT17, LAS17, ODC1, RER2, SEC7, TRX1, UGA4, VPS27 |
| cell cycle | 6 genes, or 8.7% | DAD1, DUO1, FIN1, SCC2, SPS4, SWM1 |
| transcription | 5 genes, or 7.2% | ARG82, GZF3, HPR1, PDR1, RIF1 |
| amino acid metabolic process | 2 genes, or 2.9% | ARG82, HIS2 |
| signal transduction | 2 genes, or 2.9% | COS111, RCN1 |
| can not be mapped to a GO slim term | 1 gene, or 1.4% | YDL038C |

**Table S7. Classification of FTI down-regulated genes according to biological processes**

|  |  |  |
| --- | --- | --- |
| **Biological Process** | **Frequency (%)** | **Genes Names** |
| cellular process | 25 genes, or 71.4% | CDC54, CSE1, CTF13, FAR7, FYV10, HAP5, HMI1, HUL4, IRE1, MDJ2, MET18, OSH3, PET122, PET309, RIM15, RLP24, RPA135, SNF2, SPR1, SSP2, TEF1, YJL113W, YJR141W, YPT53, ZIP2 |
| metabolic process | 15 genes, or 42.9% | CDC54, CTF13, FYV10, HAP5, HMI1, HUL4, IRE1, MET18, PET122, PET309, RIM15, RPA135, SNF2, TEF1, YJR141W |
| biological process unknown | 8 genes, or 22.9% | SPG3, YBR113W, YGR219W, YLR358C, YLR374C, YML009C-A, YPL264C, YPR126C |
| transport | 6 genes, or 17.1% | CSE1, MDJ2, OSH3, TEF1, YKR078W, YPT53 |
| cell cycle | 3 genes, or 8.6% | FAR7, RIM15, ZIP2 |
| transcription | 3 genes, or 8.6% | HAP5, MET18, RPA135 |
| amino acid metabolic process | 1 gene, or 2.9% | MET18 |
| signal transduction | 1 gene, or 2.9% | IRE1 |
| can not be mapped to a GO slim term | 1 gene, or 2.9% | YCR102C |

**Table S8. List of up-regulated genes in *Δram1* cells**

|  | |  |  |  |  |  |
| --- | --- | --- | --- | --- | --- | --- |
| **Name** | **log_2_ ratio Treated/**  **Control** | **p-value** |  | **Name** | **log_2_ ratio Treated/**  **Control** | **p-value** |
| **UpRegulated** | |  |  |  |  |  |
| YGR248W | 3.227 | 0.060016 |  | YDR031W | 0.813 | 0.133336 |
| YGR088W | 2.389 | 0.017513 |  | YBL022C | 0.804 | 0.173068 |
| YOR382W | 1.995 | 0.001006 |  | YIL113W | 0.787 | 0.142736 |
| YGR043C | 1.9 | 0.084841 |  | YGL091C | 0.78 | 0.075706 |
| YMR250W | 1.701 | 0.182348 |  | YDR349C | 0.774 | 0.146929 |
| YDR074W | 1.686 | 0.088832 |  | YJL161W | 0.771 | 0.126242 |
| YML128C | 1.677 | 0.178568 |  | YGL086W | 0.765 | 0.017386 |
| YGL119W | 1.446 | 0.029175 |  | YGL100W | 0.761 | 0.0123 |
| YNL128W | 1.4 | 0.156583 |  | YEL024W | 0.749 | 0.104133 |
| YLR252W | 1.385 | 0.097334 |  | YGL059W | 0.749 | 0.061399 |
| YLR142W | 1.282 | 0.010316 |  | YGL127C | 0.749 | 0.102733 |
| YGL121C | 1.267 | 0.021758 |  | YBR139W | 0.739 | 0.134353 |
| YML100W | 1.264 | 0.156273 |  | YGL062W | 0.728 | 0.074688 |
| YGL081W | 1.196 | 0.047694 |  | YGL087C | 0.72 | 0.1192 |
| YDL021W | 1.115 | 0.116623 |  | YGL043W | 0.718 | 0.03285 |
| YGL096W | 1.1 | 0.001565 |  | YJL164C | 0.715 | 0.105125 |
| YLR178C | 1.068 | 0.169703 |  | YCR012W | 0.715 | 0.081428 |
| YBL020W | 1.052 | 0.156873 |  | YDL204W | 0.714 | 0.114832 |
| YGL109W | 1.038 | 0.103554 |  | YGL142C | 0.713 | 0.003994 |
| YGL155W | 1.004 | 0.107585 |  | YER101C | 0.708 | 0.174492 |
| YDL022W | 0.993 | 0.172213 |  | YGL145W | 0.705 | 0.093752 |
| YDR012W | 0.988 | 0.072392 |  | YGL148W | 0.704 | 0.044408 |
| YBR105C | 0.986 | 0.028837 |  | YLR374C | 0.703 | 0.001348 |
| YOR289W | 0.98 | 0.139169 |  | YMR181C | 0.7 | 0.073453 |
| YAL040C | 0.98 | 0.138148 |  | YLR286C | 0.692 | 0.112976 |
| YDR353W | 0.971 | 0.084252 |  | YCR061W | 0.686 | 0.0342 |
| YGL152C | 0.968 | 0.038577 |  | YGL104C | 0.684 | 0.036453 |
| YNL015W | 0.962 | 0.182722 |  | YBL028C | 0.683 | 0.190981 |
| YBR157C | 0.948 | 0.042419 |  | YCL036W | 0.683 | 0.163322 |
| YGL157W | 0.947 | 0.123494 |  | YDR032C | 0.68 | 0.180104 |
| YGL071W | 0.928 | 0.024197 |  | YGL097W | 0.68 | 0.085124 |
| YER038C | 0.921 | 0.108033 |  | YAL054C | 0.679 | 0.130848 |
| YGL079W | 0.914 | 0.009372 |  | YHL021C | 0.679 | 0.139986 |
| YGL057C | 0.901 | 0.051856 |  | YLR238W | 0.673 | 0.055525 |
| YKL096W | 0.901 | 0.159348 |  | YGL067W | 0.66 | 0.049307 |
| YGL117W | 0.895 | 0.025712 |  | YOR230W | 0.658 | 0.106279 |
| YGL041C | 0.888 | 0.026429 |  | YLL056C | 0.649 | 0.049267 |
| YGL047W | 0.879 | 0.000642 |  | YMR148W | 0.643 | 0.048227 |
| YCL038C | 0.87 | 0.027108 |  | YGL147C | 0.634 | 0.029964 |
| YGL072C | 0.865 | 0.006809 |  | YGR079W | 0.634 | 0.096678 |
| YGL143C | 0.851 | 0.089337 |  | YGL139W | 0.629 | 0.017728 |
| YBL064C | 0.835 | 0.106547 |  | YGL130W | 0.628 | 0.15947 |
| YGL110C | 0.829 | 0.002145 |  | YOR136W | 0.625 | 0.009671 |
| YGR279C | 0.818 | 0.122648 |  | YOL157C | 0.624 | 0.061779 |
| YGL108C | 0.815 | 0.004817 |  | YPL219W | 0.624 | 0.136637 |

|  |  |  |
| --- | --- | --- |
| YGL105W | 0.62 | 0.181794 |
| YMR319C | 0.62 | 0.019894 |
| YHR001W-A | 0.619 | 0.079021 |
| YKR013W | 0.607 | 0.191055 |
| YGL106W | 0.603 | 0.130702 |
| YGL136C | 0.603 | 0.197715 |
| YMR244C-A | 0.602 | 0.063968 |
| YGL099W | 0.598 | 0.096897 |
| YDR347W | 0.591 | 0.154772 |
|  |  |  |

**List of down-regulated genes in *Δ ram1* cells**

| **Name** | **log_2_ ratio Treated/**  **Control** | **p-value** |  | **Name** | **log_2_ ratio Treated/**  **Control** | **p-value** |
| --- | --- | --- | --- | --- | --- | --- |
| **DownRegulated** | |  |  |  |  |  |
|  |  |  |  |  |  |  |
| YDL090C | -3.742 | 0.020063 |  | YDR314C | -0.693 | 0.128232 |
| YHR056C | -1.596 | 0.17958 |  | YHR157W | -0.689 | 0.161356 |
| YOL058W | -1.48 | 0.007202 |  | YKR054C | -0.687 | 0.141358 |
| YBL108W | -1.449 | 0.063434 |  | YGR144W | -0.658 | 0.027544 |
| YBR117C | -1.269 | 0.16758 |  | YHR216W | -0.651 | 0.075532 |
| YOL101C | -1.085 | 0.030824 |  | YLR249W | -0.64 | 0.009981 |
| YMR045C | -0.934 | 0.0984 |  | YNL007C | -0.639 | 0.042543 |
| YPR200C | -0.919 | 0.016071 |  | YDR541C | -0.633 | 0.003156 |
| YOL117W | -0.886 | 0.160428 |  | YDR248C | -0.622 | 0.015749 |
| YGR234W | -0.845 | 0.092992 |  | YJL095W | -0.621 | 0.180329 |
| YER015W | -0.83 | 0.180625 |  | YFL-TYB | -0.619 | 0.058817 |
| YDR478W | -0.83 | 0.135105 |  | YGL171W | -0.618 | 0.026931 |
| YDR538W | -0.792 | 0.184336 |  | YJR115W | -0.612 | 0.117513 |
| YER138C | -0.774 | 0.191293 |  | YIL104C | -0.6 | 0.063508 |
| YBR076W | -0.772 | 0.088363 |  | YOL056W | -0.592 | 0.013125 |
| YDR389W | -0.758 | 0.038507 |  | YNR072W | -0.592 | 0.102314 |
| YNR060W | -0.723 | 0.189085 |  | YKR094C | -0.59 | 0.187158 |
| YLR334C | -0.721 | 0.118673 |  | YKL182W | -0.589 | 0.051399 |
| YPR071W | -0.713 | 0.100266 |  | YPL238C | -0.589 | 0.052778 |

**Table S9. Classification of *Δram1* up-regulated genes according to compartment**

| **Compartment** | **Frequency (%)** | **Genes Names** |
| --- | --- | --- |
| cytoplasm | 63 genes, or 63.6% | ABC1, ACS1, AFT1, ALG13, ARC1, ARO2, AST2, ATG22, CTS1, CTT1, CUE3, FAR10, FLC3, FMP12, FMP33, GAD1, GPD1, GPM2, IDH2, KRE29, LSG1, MIC14, MMS2, MRF1, MRM2, MRP1, MSC1, NBP35, NPY1, PBI2, PGK1, PIM1, PRX1, PRY2, PST2, PUT1, PYC1, QCR10, RFT1, RIP1, RPL4B, RPL9A, RTN2, SDP1, SOL4, TFS1, TIP20, TPK1, TPS2, TRR1, TSL1, VID24, VPS73, YBL028C, YBR139W, YCR061W, YGL057C, YGL059W, YGL079W, YGL157W, YMR244C-A, YOR289W, YPS7 |
| nucleus | 24 genes, or 24.2% | AFT1, ALG13, CEG1, CLN3, CTS1, DST1, KRE29, MAD1, MIC14, MMS2, NBP35, NQM1, SDP1, SEH1, SOH1, SOL4, SRM1, TOS8, TPK1, WTM1, YBL028C, YGL157W, YMR244C-A, YOR289W |
| mitochondrion | 22 genes, or 22.2% | ABC1, ACS1, FLC3, FMP12, FMP33, IDH2, MIC14, MRF1, MRM2, MRP1, MSC1, PGK1, PIM1, PRX1, PST2, PUT1, QCR10, RIP1, TPS2, VPS73, YGL057C, YGL059W |
| cellular component unknown | 17 genes, or 17.2% | GFD2, GPG1, ICS2, TEP1, YGL041C, YGL072C, YGL081W, YGL108C, YGL109W, YGL117W, YGL152C, YGR079W, YLL056C, YLR252W, YLR374C, YMR181C, YOL157C |
| endoplasmic reticulum | 9 genes, or 9.1% | ALG13, CTS1, FAR10, FLC3, MSC1, RFT1, RTN2, TIP20, YPS7 |
| cytosol | 8 genes, or 8.1% | ACS1, ALG13, GPD1, GPM2, PYC1, RPL4B, RPL9A, SOL4 |
| can not be mapped to a GO slim term | 9 genes, or 9.1% | CDC43, CWP1, FET4, FIT2, GPI10, MLC1, PCL8, SCW4, YMR148W |

**Table S10. Classification of *Δram1* up-regulated genes according to biological processes**

| **Biological Process** | **Frequency (%)** | **Genes Names** |
| --- | --- | --- |
| cellular process | 62 genes, or 62.6% | ABC1, ACS1, AFT1, ALG13, ARC1, ARO2, ATG22, CDC43, CEG1, CLN3, CTS1, CTT1, CWP1, DST1, FAR10, FET4, GAD1, GPD1, GPG1, GPI10, IDH2, KRE29, LSG1, MAD1, MLC1, MMS2, MRF1, MRM2, MRP1, MSC1, NBP35, NPY1, PBI2, PCL8, PGK1, PIM1, PRX1, PUT1, PYC1, QCR10, RFT1, RIP1, RPL4B, RPL9A, SCW4, SDP1, SEH1, SOH1, SOL4, SRM1, TEP1, TFS1, TIP20, TOS8, TPK1, TRR1, VID24, VPS73, WTM1, YBL028C, YBR139W, YCR061W |
| metabolic process | 46 genes, or 46.5% | ABC1, ACS1, AFT1, ALG13, ARC1, ARO2, CDC43, CEG1, CTS1, CTT1, DST1, GAD1, GPD1, GPI10, IDH2, KRE29, MMS2, MRF1, MRM2, MRP1, MSC1, NBP35, NPY1, PBI2, PCL8, PGK1, PIM1, PRX1, PUT1, PYC1, QCR10, RFT1, RIP1, RPL4B, RPL9A, SOH1, SOL4, TEP1, TFS1, TOS8, TPK1, TPS2, TRR1, TSL1, VID24, YBR139W |
| biological process unknown | 32 genes, or 32.3% | AST2, CUE3, FMP12, FMP33, GFD2, GPM2, ICS2, MIC14, NQM1, PRY2, PST2, RTN2, YGL041C, YGL057C, YGL059W, YGL072C, YGL079W, YGL081W, YGL108C, YGL109W, YGL117W, YGL152C, YGL157W, YGR079W, YLR252W, YLR374C, YMR148W, YMR181C, YMR244C-A, YOL157C, YOR289W, YPS7 |
| transport | 16 genes, or 16.2% | AFT1, ARC1, ATG22, FET4, FIT2, FLC3, LSG1, MAD1, MLC1, RFT1, SEH1, SRM1, TIP20, VID24, VPS73, WTM1 |
| cell cycle | 8 genes, or 8.1% | CLN3, DST1, FAR10, MAD1, MLC1, MSC1, TOS8, WTM1 |
| amino acid metabolic process | 5 genes, or 5.1% | ARC1, ARO2, GAD1, IDH2, PUT1 |
| transcription | 5 genes, or 5.1% | AFT1, CEG1, DST1, SOH1, TOS8 |
| signal transduction | 5 genes, or 5.1% | CDC43, GPG1, SDP1, TEP1, TPK1 |
| can not be mapped to a GO slim term | 1 gene, or 1.0% | YLL056C |

**Table S11. Classification of *Δram1* down-regulated genes according to biological processes**

| **Biological Process** | **Frequency (%)** | **Genes Names** |
| --- | --- | --- |
| cellular process | 26 genes, or 68.4% | ARG1, BCK1, DYN1, ECM8, FAA2, FAS1, FRE4, IMD2, IZH4, PAD1, RAD34, RAM1, REC104, ROK1, RPL40B, RRI2, RSC30, SAC7, SHQ1, SIS1, SNM1, THI4, TKL2, YEF3, YER138C, YMR045C |
| metabolic process | 20 genes, or 52.6% | ARG1, BCK1, FAA2, FAS1, IMD2, IZH4, PAD1, RAD34, RAM1, REC104, ROK1, RPL40B, RRI2, RSC30, SHQ1, SIS1, SNM1, THI4, TKL2, YEF3 |
| biological process unknown | 8 genes, or 21.1% | GPM3, YBL108W, YDR248C, YDR541C, YJR115W, YLR334C, YPL238C, YPR071W |
| signal transduction | 3 genes, or 7.9% | BCK1, RRI2, SAC7 |
| transport | 3 genes, or 7.9% | DYN1, FRE4, HXT17 |
| cell cycle | 3 genes, or 7.9% | DYN1, REC104, SAC7 |
| amino acid metabolic process | 1 gene, or 2.6% | ARG1 |
| transcription | 1 gene, or 2.6% | RSC30 |
| can not be mapped to a GO slim term | 2 genes, or 5.3% | ARR2, YHB1 |
| not yet annotated | 1 gene, or 2.6% | YFL-TyB |

**Table S12. List of up-regulated genes in GGTI-298 treated cells**

| **Name** | **log_2_ ratio Treated/**  **Control** | **p-value** |  | **Name** | **log_2_ ratio Treated/**  **Control** | p-value |
| --- | --- | --- | --- | --- | --- | --- |
| **UpRegulated** | |  |  |  |  |  |
| YBR099C | 1.363 | 0.004542 |  | YBR090C-A | 0.671 | 0.009013 |
| YGL072C | 1.124 | 0.002358 |  | YHR155W | 0.67 | 0.026996 |
| YLR161W | 0.946 | 0.116274 |  | YNL235C | 0.669 | 0.029978 |
| YDL016C | 0.943 | 0.014385 |  | YIL060W | 0.669 | 0.108147 |
| YDR461W | 0.941 | 0.065368 |  | YOL135C | 0.668 | 0.162254 |
| YHR049C-A | 0.919 | 0.010294 |  | YGR294W | 0.661 | 0.052849 |
| YKL123W | 0.892 | 0.00677 |  | YBR201W | 0.66 | 0.179735 |
| YDR340W | 0.884 | 0.004594 |  | YGR075C | 0.659 | 0.014264 |
| YHR145C | 0.882 | 0.044402 |  | YLR294C | 0.659 | 0.146505 |
| YOR258W | 0.855 | 0.059412 |  | YJL142C | 0.653 | 0.058269 |
| YKL076C | 0.842 | 0.050197 |  | YIL073C | 0.652 | 0.107953 |
| YPL001W | 0.841 | 0.009994 |  | YDL108W | 0.651 | 0.005134 |
| YDR157W | 0.831 | 0.09344 |  | YBR102C | 0.644 | 0.014137 |
| YBL005W-B | 0.826 | 0.154417 |  | YFR039C | 0.644 | 0.176158 |
| YCR041W | 0.819 | 0.021731 |  | YPL271W | 0.64 | 0.181975 |
| YDR366C | 0.809 | 0.069296 |  | YPL058C | 0.639 | 0.045767 |
| YGR182C | 0.805 | 0.055665 |  | YDL064W | 0.638 | 0.002105 |
| YPL068C | 0.8 | 0.012675 |  | YDR457W | 0.637 | 0.113906 |
| YOL106W | 0.797 | 0.010272 |  | YPR067W | 0.632 | 0.049321 |
| YJR162C | 0.795 | 0.166432 |  | YMR003W | 0.632 | 0.029928 |
| YNR077C | 0.794 | 0.165403 |  | YIL001W | 0.631 | 0.013786 |
| YLL051C | 0.786 | 0.032736 |  | YHL046C | 0.631 | 0.034044 |
| YNL234W | 0.785 | 0.047989 |  | YCR049C | 0.63 | 0.18186 |
| YGR035C | 0.782 | 0.005518 |  | YEL033W | 0.629 | 0.11772 |
| YIR005W | 0.773 | 0.028547 |  | YCL058C | 0.625 | 0.108976 |
| YCL056C | 0.773 | 0.037609 |  | YLR334C | 0.625 | 0.041295 |
| YNL337W | 0.771 | 0.054263 |  | YDR005C | 0.62 | 0.084427 |
| YKL066W | 0.769 | 0.026346 |  | YBR206W | 0.616 | 0.029618 |
| YNL319W | 0.758 | 0.140722 |  | YKL225W | 0.614 | 0.136456 |
| YER109C | 0.754 | 0.000563 |  | YPL046C | 0.604 | 0.099803 |
| YBL077W | 0.754 | 0.003813 |  | YGL136C | 0.603 | 0.015687 |
| YIL061C | 0.752 | 0.005884 |  | YJL015C | 0.602 | 0.00284 |
| YMR087W | 0.743 | 0.007474 |  | YJL153C | 0.6 | 0.028431 |
| YMR210W | 0.734 | 0.029847 |  | YOL150C | 0.599 | 0.011707 |
| YOR121C | 0.727 | 0.103918 |  | YLR095C | 0.598 | 0.010774 |
| YMR141C | 0.713 | 0.022838 |  | YLR099C | 0.598 | 0.004051 |
| YOR152C | 0.701 | 0.032346 |  | YOL151W | 0.598 | 0.005354 |
| YMR302C | 0.691 | 0.057978 |  | YOR050C | 0.593 | 0.092865 |
| YIL086C | 0.689 | 0.030561 |  | YJL152W | 0.592 | 0.061799 |
| YFR029W | 0.686 | 0.007383 |  | YIL176C | 0.592 | 0.039134 |
| YBR046C | 0.686 | 0.199557 |  | YLR392C | 0.589 | 0.042087 |
| YDL140C | 0.679 | 0.113854 |  | YGL032C | 0.588 | 0.096706 |
| YNL170W | 0.674 | 0.073689 |  | YOR300W | 0.585 | 0.15469 |

**List of down-regulated genes in GGTI-298 treated cells**

| **Name** | **log_2_ ratio Treated/**  **Control** | | | **p-value** | |  | **Name** | **log_2_ ratio Treated/**  **Control** | **p-value** |
| --- | --- | --- | --- | --- | --- | --- | --- | --- | --- |
| **DownRegulated** | | | |  | |  |  |  |  |
| YDR077W | -1.885 | | | 0.03647 | |  | YJR077C | -0.764 | 0.034529 |
| YLR339C | -1.578 | | | 0.126962 | |  | YJR047C | -0.755 | 0.109319 |
| YHR175W | -1.452 | | | 0.042561 | |  | YPR053C | -0.733 | 0.082394 |
| YAR073W | -1.368 | | | 0.063457 | |  | YMR175W | -0.731 | 0.070642 |
| YJL159W | -1.338 | | | 0.027675 | |  | YLR027C | -0.724 | 0.085076 |
| YNL190W | -1.336 | | | 0.136769 | |  | YJL062W | -0.718 | 0.077691 |
| YOR086C | -1.238 | | | 0.137547 | |  | YGL114W | -0.707 | 0.109276 |
| YDL046W | -1.196 | | | 0.045988 | |  | YPR149W | -0.702 | 0.086501 |
| YGL155W | -1.195 | | | 0.084083 | |  | YPR032W | -0.697 | 0.12253 |
| YKR044W | -1.16 | | | 0.185787 | |  | YHR216W | -0.685 | 0.11178 |
| YOR331C | -1.15 | | | 0.119006 | |  | YIL138C | -0.679 | 0.09203 |
| YPL135W | -1.143 | | | 0.026537 | |  | YER126C | -0.676 | 0.082337 |
| YPL036W | -1.092 | | | 0.18387 | |  | YGL040C | -0.676 | 0.184377 |
| YHR088W | -1.068 | | | 0.197971 | |  | YDR033W | -0.673 | 0.002578 |
| YJR121W | -1.005 | | | 0.07692 | |  | YDR328C | -0.665 | 0.089626 |
| YKR051W | -0.999 | | | 0.118446 | |  | YJR002W | -0.663 | 0.052713 |
| YAL005C | -0.965 | | | 0.114975 | |  | YLR342W | -0.66 | 0.02061 |
| YKL164C | -0.965 | | | 0.009599 | |  | YGR279C | -0.654 | 0.065494 |
| YCL009C | -0.965 | | | 0.081785 | |  | YGL028C | -0.65 | 0.164845 |
| YKL104C | -0.947 | | | 0.083457 | |  | YPL015C | -0.65 | 0.019102 |
| YJL005W | -0.931 | | | 0.094208 | |  | YDR492W | -0.635 | 0.037865 |
| YKL163W | -0.914 | | | 0.065441 | |  | YJR139C | -0.634 | 0.084172 |
| YIL123W | -0.908 | | | 0.033948 | |  | YGR138C | -0.631 | 0.044915 |
| YHR128W | -0.899 | | | 0.137807 | |  | YNL066W | -0.631 | 0.051174 |
| YDR134C | -0.893 | | | 0.086517 | |  | YMR215W | -0.629 | 0.011669 |
| YPR181C | -0.884 | | | 0.012738 | |  | YPL028W | -0.629 | 0.157208 |
| YJL012C | -0.88 | | | 0.138438 | |  | YGR234W | -0.625 | 0.075959 |
| YOR014W | -0.865 | | | 0.104799 | |  | YOL136C | -0.625 | 0.029055 |
| YOL059W | -0.862 | | | 0.013292 | |  | YNL046W | -0.624 | 0.10524 |
| YPL226W | -0.86 | | | 0.017118 | |  | YDR211W | -0.62 | 0.142626 |
| YKL013C | -0.85 | | | 0.026956 | |  | YLR048W | -0.619 | 0.116741 |
| YHR133C | -0.847 | | | 0.086498 | |  | YHL048W | -0.615 | 0.108667 |
| YKL028W | -0.835 | | | 0.175831 | |  | YNL104C | -0.615 | 0.005397 |
| YGR250C | -0.835 | | | 0.165991 | |  | YDL122W | -0.613 | 0.086742 |
| YDR038C | -0.828 | | | 0.184715 | |  | YJL178C | -0.611 | 0.096303 |
| YGL008C | -0.827 | | | 0.069076 | |  | YKL035W | -0.61 | 0.152043 |
| YMR056C | -0.817 | | | 0.093766 | |  | YMR297W | -0.61 | 0.008289 |
| YLR083C | -0.808 | | | 0.165588 | |  | YDL015C | -0.607 | 0.00704 |
| YHR174W | -0.802 | | | 0.153439 | |  | YPR028W | -0.606 | 0.003 |
| YMR203W | -0.8 | | | 0.009592 | |  | YOR123C | -0.605 | 0.068602 |
| YGR111W | -0.8 | | | 0.092164 | |  | YGL244W | -0.604 | 0.171125 |
| YPR016C | -0.791 | | | 0.030982 | |  | YKR028W | -0.603 | 0.181764 |
| YOR240W | -0.788 | | | 0.053847 | |  | YGL082W | -0.594 | 0.059144 |
| YLR016C | -0.784 | | | 0.032014 | |  | YHL003C | -0.593 | 0.002224 |
| YML093W | -0.779 | | | 0.084313 | |  | YGL213C | -0.59 | 0.114932 |
| YMR079W | -0.775 | | | 0.117524 | |  | YIR024C | -0.59 | 0.054078 |
| YJL123C | -0.765 | | | 0.077327 | |  | YER005W | -0.587 | 0.164261 |
| YDL077C | | -0.585 | 0.097685 | |  | | | | |

| **Compartment** | **Frequency (%)** | **Genes Names** |
| --- | --- | --- |
| cellular component unknown | 47 genes, or 54.7% | FYV5, ICT1, IRC9, PAU12, PAU13, PAU14, PSY1, YBL077W, YBR099C, YBR206W, YCR041W, YCR049C, YDL016C, YDR157W, YDR340W, YDR366C, YEL033W, YFR039C, YGL072C, YGR035C, YGR182C, YHR049C-A, YHR145C, YIL060W, YIL086C, YJL015C, YJL152W, YJR162C, YKL066W, YKL123W, YKL225W, YLR161W, YLR294C, YLR334C, YMR087W, YMR141C, YMR210W, YNL170W, YNL235C, YNL319W, YNL337W, YNR077C, YOL106W, YOL150C, YOR050C, YOR121C, YOR300W |
| cytoplasm | 20 genes, or 23.3% | ATP15, DER1, EXO84, FLO8, FRE6, GRE2, HAT1, HNT3, INO1, ISA2, MRM2, RPO21, YCL056C, YIL001W, YLR392C, YME2, YMR003W, YNL234W, YSP1, ZTA1 |
| nucleus | 20 genes, or 23.3% | ELC1, FLO8, GRE2, HAT1, HNT3, IOC2, IST3, KIN28, MAF1, MED7, NHP6B, PRP38, RPO21, SNP1, SPO22, TOM1, UBC9, YBL005W-B, YPL068C, ZTA1 |
| mitochondrion | 7 genes, or 8.1% | ATP15, ISA2, MRM2, RPO21, YME2, YMR003W, YSP1 |
| endoplasmic reticulum | 1 gene, or 1.2% | DER1 |
| can not be mapped to a GO slim term | 5 genes, or 5.8% | AGA2, MFA1, PDR12, PTR3, YOR152C |

**Table S13. Classification of GGTI-298 up-regulated genes according to compartment**

**Table S14. Classification of GGTI-298 up-regulated genes according to biological processes**

| **Biological Process** | **Frequency (%)** | **Genes Names** |
| --- | --- | --- |
| biological process unknown | 48 genes, or 55.8% | YBL077W, YBR099C, YBR206W, YCL056C, YCR041W, YCR049C, YDL016C, YDR157W, YDR340W, YDR366C, MTC7, YFR039C, YGL072C, YGR035C, YGR182C, PAU12, PAU13, YHR049C-A, YHR145C, YSP1, YIL001W, YIL060W, YIL086C, PAU14, YJL015C, YJL152W, YJR162C, YKL066W, PSY1, YKL123W, YKL225W, YLR161W, YLR334C, YLR392C, AIM34, YMR141C, YNL170W, YNL235C, YNL319W, YNL337W, YNR077C, YOL106W, YOL150C, YOR050C, YOR121C, YOR152C, HNT3, YPL068C |
| cellular process | 34 genes, or 39.5% | YBL005W-B, NHP6B, EXO84, DER1, FYV5, UBC9, KIN28, RPO21, MAF1, TOM1, MFA1, FLO8, AGA2, MRM2, PRP38, SNP1, SPO22, IST3, IRC9, FRE6, IOC2, ICT1, YLR294C, YMR087W, YMR210W, YME2, MED7, GRE2, YOR300W, HAT1, ELC1, PDR12, ATP15, ISA2 |
| metabolic process | 26 genes, or 30.2% | NHP6B, EXO84, DER1, UBC9, KIN28, RPO21, MAF1, TOM1, FLO8, MRM2, PRP38, SNP1, IST3, IRC9, INO1, ICT1, YLR294C, YMR087W, YMR210W, MED7, GRE2, HAT1, ELC1, PDR12, ATP15, ISA2 |
| transcription | 8 genes, or 9.3% | NHP6B, KIN28, RPO21, MAF1, FLO8, MED7, HAT1, ELC1 |
| transport | 7 genes, or 8.1% | EXO84, TOM1, IST3, FRE6, PDR12, ATP15, ISA2 |
| cell cycle | 4 genes, or 4.7% | UBC9, TOM1, SPO22, YOR300W |
| signal transduction | 1 gene, or 1.2% | MFA1 |
| cellular amino acid metabolic process | 0 genes, or 0% | None |
| Other | 3 genes, or 3.5% | ZTA1, PTR3, YNL234W |

**Table S15. Classification of nuclear-located GGTI-298 up-regulated genes according to biological process**

| **Biological Process** | **Frequency (%)** | **Frequency, on the whole genome, of genes belonging to each Biological Process** | **P-value** | **Genes Names** |
| --- | --- | --- | --- | --- |
| transcription, DNA-dependent | 8 genes, or 40.0% | 552 genes, or 7.7% | 0.008 | NHP6B, KIN28, RPO21, MAF1, FLO8, MED7, HAT1, ELC1 |
| RNA biosynthetic process | 8 genes, or 40.0% | 554 genes, or 7.7% | 0.008 | NHP6B, KIN28, RPO21, MAF1, FLO8, MED7, HAT1, ELC1 |
| RNA metabolic process | 11 genes, or 55.0% | 1151 genes, or 16.1% | 0.009 | NHP6B, KIN28, RPO21, MAF1, FLO8, PRP38, SNP1, IST3, MED7, HAT1, ELC1 |

**Table S16. Classification of GGTI-298 down-regulated genes according to compartment**

| **Compartment** | **Frequency (%)** | **Genes Names** |
| --- | --- | --- |
| cytoplasm | 65 genes, or 68.4% | AAC1, AAT2, ANB1, ATG27, ATP2, CTR2, CYR1, EMP70, ENO2, ERG10, FKS1, GCD6, GPD2, HEM2, HOM6, HST2, ILV6, IMD2, ISU1, IZH1, LAG1, LAS21, LEU4, MIR1, MRH1, NCE102, NEW1, NPC2, NSA2, NSG1, PFK27, PMA1, PMA2, PML1, PRC1, RPS0B, RTS1, SAP190, SEC14, SEC23, SED1, SKI8, SKP1, SRO7, SSA1, SUN4, TCB1, TFA1, TIF6, TOM40, TPM2, TPO2, TSC13, UBP1, UGP1, VAM6, VTC4, YGL082W, YGR111W, YGR250C, YHB1, YIR024C, YJL123C, YND1, YNL046W |
| Mitochondrion | 24 genes, or 25.3% | AAC1, ATG27, ATP2, CYR1, ENO2, FKS1, GPD2, ILV6, ISU1, LEU4, MIR1, MRH1, NCE102, NEW1, PMA1, PMA2, SED1, SUN4, TCB1, TFA1, TOM40, TSC13, YHB1, YIR024C |
| nucleus | 19 genes, or 20% | COS8, HEM2, HOM6, LEO1, MPP10, NSA2, PML1, RPF1, RTF1, RTS1, SKI8, SKP1, SSA1, TFA1, TIF6, UIP5, UTP14, YGL082W, YGR111W |
| endoplasmic reticulum | 10 genes, or 10.5% | IZH1, LAG1, LAS21, NCE102, NSG1, PRC1, TSC13, UBP1, VTC4, YNL046W |
| cytosol | 8 genes, or 8.4% | ENO2, ERG10, GPD2, RPS0B, SEC14, SRO7, SSA1, YHB1 |
| cellular component unknown | 6 genes, or 6.3% | GFA1, IMD1, YKR051W, YLR339C, YOR331C, YPR053C |
| can not be mapped to a GO slim term | 16 genes, or 16.8% | ARC19, CDC43, ENA5, FUR1, GAS3, HSP150, PIR1, PIR3, SCW11, SCW4, SIM1, SIP18, YDR134C, YGL114W, YNL190W, YOP1 |
| not yet annotated | 1 gene, or 1.1% | YOR240W |

**Table S17. Classification of nuclear-located GGTI-298 down-regulated genes according to biological process**

**Table S18. Classification of endoplasmic reticulum-located GGTI-298 down-regulated genes according to biological process**

| **Biological Process** | **Frequency (%)** | **Frequency, on the whole genome, of genes belonging to each Biological Process** | **P-value** | **Genes Names** |
| --- | --- | --- | --- | --- |
| maturation of 5.8S rRNA from tricistronic rRNA transcript (SSU-rRNA, 5.8S rRNA, LSU-rRNA) | 4 genes, or 21.1% | 34 background genes, or 0.5% | 0.0004 | NSA2, RPF1, UTP14, TIF6 |
| maturation of 5.8S rRNA | 4 genes, or 21.1% | 36 background genes, or 0.5% | 0.0005 | NSA2, RPF1, UTP14, TIF6 |
| maturation of LSU-rRNA from tricistronic rRNA transcript (SSU-rRNA, 5.8S rRNA, LSU-rRNA) | 3 genes, or 15.8% | 14 background genes, or 0.2% | 0.0015 | NSA2, RPF1, TIF6 |
| maturation of LSU-rRNA | 3 genes, or 15.8% | 14 background genes, or 0.2% | 0.0015 | NSA2, RPF1, TIF6 |

| **Biological Process** | **Frequency (%)** | **Frequency, on the whole genome, of genes belonging to each Biological Process** | **P-value** | **Genes Names** |
| --- | --- | --- | --- | --- |
| lipid metabolic process | 5 genes, or 50.0% | 235 background genes, or 3.2% | 8E-04 | TSC13, IZH1, LAG1, NSG1, LAS21 |

**Table S19. Classification of GGTI-298 down-regulated genes according to biological processes**

| **Biological Process** | **Frequency (%)** | **Genes Names** |
| --- | --- | --- |
| cellular process | 68 genes, or 71.6% | SSA1, ILV6, TSC13, NPC2, VAM6, UBP1, ENA5, SED1, GCD6, SKP1, IZH1, YND1, NSA2, PMA1, SCW11, HEM2, CDC43, SKI8, RTF1, LAG1, RPF1, FUR1, NSG1, ENO2, CTR2, IMD2, TPM2, CYR1, VTC4, LAS21, HSP150, ATG27, MPP10, ANB1, ATP2, HOM6, ARC19, TFA1, UGP1, GFA1, PIR3, PIR1, SAP190, PML1, AAT2, RPS0B, EMP70, FKS1, UTP14, AAC1, SEC14, TOM40, PRC1, SUN4, LEU4, GPD2, PFK27, RTS1, LEO1, HST2, ERG10, ISU1, NEW1, TIF6, YOP1, SRO7, NCE102, SEC23 |
| metabolic process | 50 genes, or 52.6% | SSA1, ILV6, TSC13, VAM6, UBP1, GCD6, SKP1, IZH1, YND1, NSA2, HEM2, CDC43, SKI8, RTF1, LAG1, RPF1, FUR1, NSG1, ENO2, IMD2, VTC4, LAS21, ATG27, MPP10, ANB1, ATP2, HOM6, TFA1, UGP1, GFA1, SAP190, PML1, AAT2, RPS0B, FKS1, UTP14, AAC1, SEC14, PRC1, LEU4, GPD2, PFK27, RTS1, LEO1, HST2, ERG10, ISU1, TIF6, NCE102, SEC23 |
| Transport | 27 genes, or 28.4% | SSA1, NPC2, ENA5, PMA1, YGL114W, TPO2, RPF1, CTR2, TPM2, VTC4, ATG27, MIR1, ATP2, PIR1, PML1, RPS0B, FKS1, AAC1, SEC14, TOM40, PMA2, NEW1, TIF6, YOP1, SRO7, NCE102, SEC23 |
| biological process unknown | 17 genes, or 17.9% | IMD1, MRH1, YDR134C, YGL082W, YGR250C, COS8, SIM1, YIR024C, MTC1, UIP5, YKR051W, YLR339C, SIP18, GAS3, TCB1, YOR331C, YPR053C |
| signal transduction | 4 genes, or 4.2% | CDC43, CYR1, SAP190, SRO7 |
| cell cycle | 4 genes, or 4.2% | SKP1, SKI8, SAP190, RTS1 |
| cellular amino acid metabolic process | 4 genes, or 4.2% | ILV6, HOM6, AAT2, LEU4 |
| Transcription | 4 genes, or 4.2% | RTF1, TFA1, LEO1, HST2 |
| Other | 5 genes, or 5.3% | YGR111W, YHB1, SCW4, YNL046W, YNL190W |
| not_yet_annotated | 1 gene, or 1.1% | YOR240W |
